# Identification of amino acid domains of *Borrelia burgdorferi* P66 that are surface exposed and important for localization, oligomerization, and porin function of the protein

**DOI:** 10.3389/fcimb.2022.991689

**Published:** 2022-09-23

**Authors:** Michael W. Curtis, Christa H. Fierros, Beth L. Hahn, Matthew C. Surdel, Julie Kessler, Phillip N. Anderson, Marine Vandewalle-Capo, Mari Bonde, Jieqing Zhu, Sven Bergström, Jenifer Coburn

**Affiliations:** ^1^ Department of Microbiology and Immunology, Medical College of Wisconsin, Milwaukee, WI, United States; ^2^ Department of Medicine, Division of Infectious Diseases, Medical College of Wisconsin, Milwaukee, WI, United States; ^3^ Umeå Centre for Microbial Research, Umeå University, Umeå, Sweden; ^4^ Department of Molecular Biology, Umeå University, Umeå, Sweden; ^5^ Laboratory for Molecular Infection Medicine Sweden, Umeå University, Umeå, Sweden; ^6^ Department of Chemistry, Umeå University, Umeå, Sweden; ^7^ Blood Research Institute, Versiti, Milwaukee, WI, United States; ^8^ Department of Biochemistry, Medical College of Wisconsin, Milwaukee, WI, United States

**Keywords:** *Borrelia burgdorferi*, Lyme disease, infectious disease, P66, bacterial pathogenesis, c-Myc epitope tag

## Abstract

P66, a bifunctional integral outer membrane protein, is necessary for *Borrelia burgdorferi* to establish initial infection and to disseminate in mice. The integrin binding function of P66 facilitates extravasation and dissemination, but the role of its porin function during murine infection has not been investigated. A limitation to studying P66 porin function during mammalian infection has been the lack of structural information for P66. In this study, we experimentally characterized specific domains of P66 with regard to structure and function. First, we aligned the amino acid sequences of P66 from Lyme disease-causing *Borrelia* and relapsing fever-causing *Borrelia* to identify conserved and unique domains between these disease-causing clades. Then, we examined whether specific domains of P66 are exposed on the surface of the bacteria by introducing c-Myc epitope tags into each domain of interest. The c-Myc epitope tag inserted C-terminally to E33 (highly conserved domain), to T187 (integrin binding region domain and a non-conserved domain), and to E334 (non-conserved domain) were all detected on the surface of *Borrelia burgdorferi*. The c-Myc epitope tag inserted C-terminally to E33 and D303 in conserved domains disrupted P66 oligomerization and porin function. In a murine model of infection, the E33 and D303 mutants exhibited decreased infectivity and dissemination. Taken together, these results suggest the importance of these conserved domains, and potentially P66 porin function, *in vivo*.

## Introduction

Lyme disease (LD) is a significant public health concern in the northern hemisphere. LD is caused by a group of spirochetes called *Borrelia burgdorferi* sensu lato, which are transmitted to people by certain species of *Ixodes* ticks (Burgdorfer et al., 1982; Radolf et al., 2012). Recently and controversially, some groups have advocated changing the name to *Borreliella burgdorferi*, (Adeolu and Gupta, 2014), but we have retained the conventional name in this article. In the United States, LD is primarily caused by *Borrelia burgdorferi* and can cause mild symptoms such as erythema migrans, fever, malaise (Steere et al., 1977; Steere et al., 1983; Nadelman et al., 1996) or more severe symptoms such as neuroborreliosis, Lyme carditis, or Lyme arthritis in the large weight bearing joints (Steere et al., 1980; Steere et al., 1987; Stiernstedt et al., 1988). While there were over 23,000 confirmed cases of LD reported to the Centers for Disease Control in 2019, recent estimates suggest that the actual number of annual LD cases diagnosed and treated in the United States exceeds 450,000 (CDC, 2019; Schwartz et al., 2021; Kugeler et al., 2021). Therefore, LD is the most common arthropod-borne disease in the United States.

Investigations of how *Borrelia burgdorferi* establishes and maintains a persistent, disseminated infection have been a long-standing area of emphasis in the field. *Borrelia burgdorferi* produces outer membrane proteins that bind to and interact with host components (reviewed in: Coburn et al., 2013). Outer membrane proteins have been widely studied to examine their roles in *Borrelia burgdorferi* host colonization, dissemination, and immune evasion (reviewed in: Coburn et al., 2013; Brissette and Gaultney, 2014; Caine and Coburn, 2016). P66 is a bifunctional integral outer membrane protein that has a role in mammalian host colonization and dissemination (Ristow et al., 2012; Kumar et al., 2015; Ristow et al., 2015). *In vitro*, P66 binds to β_3_-chain integrins and at least two β_1_-chain integrins; it also forms a porin with an estimated diameter of 1.9 nm and a central constriction of 0.8 nm (Skare et al., 1997; Coburn et al., 1999; Defoe and Coburn, 2001; Coburn and Cugini, 2003; Barcena-Uribarri et al., 2013). Similar to other bacterial porins, P66 is a β-barrel protein that self-oligomerizes into a higher order complex in the bacterial outer membrane (Kenedy et al., 2014). Unlike other bacterial porins, P66 oligomerizes into a heptamer or an octamer (Barcena-Uribarri et al., 2013; Kenedy et al., 2014) instead of the traditional trimer (Welte et al., 1995). P66 is produced during mammalian infection and is one of the diagnostic antigens used for LD serology (Coleman and Benach, 1987; Dressler et al., 1993; Bunikis et al., 1996; Ntchobo et al., 2001; Nowalk et al., 2006; Theel, 2016). Interestingly, when a strain of *Borrelia burgdorferi* is unable to produce P66 (*p66::kanR*, hereinafter referred to as Δ*p66*), the bacterium is incapable of establishing infection in mice (Ristow et al., 2012).

Several studies have investigated the critical function of P66 in *Borrelia burgdorferi* mammalian infection. The integrin-binding function of P66 facilitates dissemination and extravasation (Ristow et al., 2015; Kumar et al., 2015; Curtis et al., 2018). However, integrin binding is not the critical *in vivo* function of P66 since *Borrelia burgdorferi* producing an integrin binding deficient mutant of P66 is still able to establish infection in mice (Ristow et al., 2015; Curtis et al., 2018). There is no evidence that P66 has a role in maintaining outer membrane stability because Δ*p66* is not more susceptible to detergents, shear forces, or osmotic stress compared to WT (Curtis et al., 2018). To date, there is not any evidence that P66 confers resistance to the host immune system. Δ*p66* is unable to establish infection in TLR2^-/-^, MyD88^-/-^, macrophage depleted, dendritic cell depleted, or neutrophil depleted mice, and it is not more susceptible than WT to the major skin antimicrobial peptide (i.e. mCRAMP) *in vitro* (Ristow et al., 2012; Ristow et al., 2015; Curtis et al., 2018) or to human complement (Ristow et al., 2015). Thus, the critical *in vivo* function of P66 remains unknown.

Interestingly, Δ*p66 Borrelia burgdorferi* were able to survive in the protected environment of dialysis membrane chambers inserted into rat peritoneal cavities for two weeks (Ristow et al., 2012). However, one caveat to this experiment is that the peritoneum is not a biologically relevant site of infection, although it is a useful model to evaluate changes in gene expression and protein production as compared to *in vitro* cultivation conditions (Ristow et al., 2012). Another caveat is that the dialysis membrane chamber used has a molecular weight cutoff of 8,000 Da, suggesting that this environment alters nutrient availability and interaction with immune system components relative to physiological conditions.

Currently, there is no crystal structure of P66, nor does P66 have any homologues outside of the *Borrelia* genus to aid in designing targeted mutagenesis directed at disrupting the porin function of P66. The *Borrelia* genus does include a number of species that cause a disease known as relapsing fever (RF). RF causes recurring episodes of febrile illness followed by asymptomatic periods and, with the exception of *B. miyamotoi*, is transmitted by different vector ticks and the human-body louse (reviewed in: Southern and Sanford, 1969; Dworkin et al., 2008). Since P66 is specific to the *Borrelia* genus, we aligned the amino acid sequences from nine RF-causing *Borrelia* and four LD-causing *Borrelia* species to identify amino acid domains that are conserved and unique between the two disease-causing clades. Then, through directed insertional mutagenesis, we explored whether the conserved domains and/or unique domains are exposed on the surface of *Borrelia burgdorferi* and whether the domains have a role in P66 localization, oligomerization, porin function, and infectivity.

## Materials and methods

### Bacterial strains and growth conditions

*E. coli* strains (**Supplementary Table S1**) were grown at 30°C in LB broth or on LB agar, each supplemented with 0.2% dextrose, which we had previously found optimal for *E. coli* strains harboring a plasmid containing the *p66* locus (Coburn et al., 1999). For selection of clones harboring the pBSV2G plasmid, LB broth and LB agar were supplemented with 5 μg ml^-1^ gentamicin. If spontaneous gentamicin resistant mutants appeared on control plates, antibiotic concentration was increased to 10 µg ml^-1^ for selection.

*Borrelia burgdorferi* B31 A3 (Elias et al., 2002), B313 (Sadziene et al., 1993), and derivative strains ( (Ristow et al., 2012) and **Supplementary Table S2**) were grown in Barbour-Stoenner-Kelly II (BSKII) (Barbour, 1984) medium at 33°C. *Borrelia burgdorferi* HB19 (Coburn et al., 1993) and derivative strains were grown in Modified Kelly Pettenkofer (MKP) (Preac-Mursic et al., 1986) medium at 33°C. For *Borrelia burgdorferi* growth in plates, BSKII medium was supplemented with 6.8 g l^-1^ agarose (Samuels, 1995) and incubated at 33°C with 2% CO_2_ for two weeks. For selection of colonies producing c-Myc tagged P66 complemented on the pBSV2G plasmid (*p66* ^cp^) or complemented on the chromosome (*p66* ^cc^), plates were supplemented with 40 μg ml^-1^ gentamicin. Some transformants in the HB19 background were isolated on semisolid medium, but others were isolated by limiting dilution in MKP in a 96-well plate as previously described (Yang et al., 2004; Battisti et al., 2008).

### Enumeration of bacteria

All culture density determination of *Borrelia* liquid cultures were manually determined by darkfield microscopy on a Petroff-Hausser counting chamber (Electron Microscopy Sciences).

### Prediction of P66 topology

PRED-TMBB^1^ is a web server that uses a Hidden Markov Model to predict and discriminate beta-barrel outer membrane proteins using an amino acid sequence (Bagos et al., 2004a; Bagos et al., 2004b). It was used to predict the surface exposed regions of the mature (AA sequence minus the 21 amino acid secretion signal sequence) P66 protein from *Borrelia burgdorferi* B31 (NP_212737.1) (Fraser et al., 1997).

### Introduction of c-Myc tags

**Figure 1:**
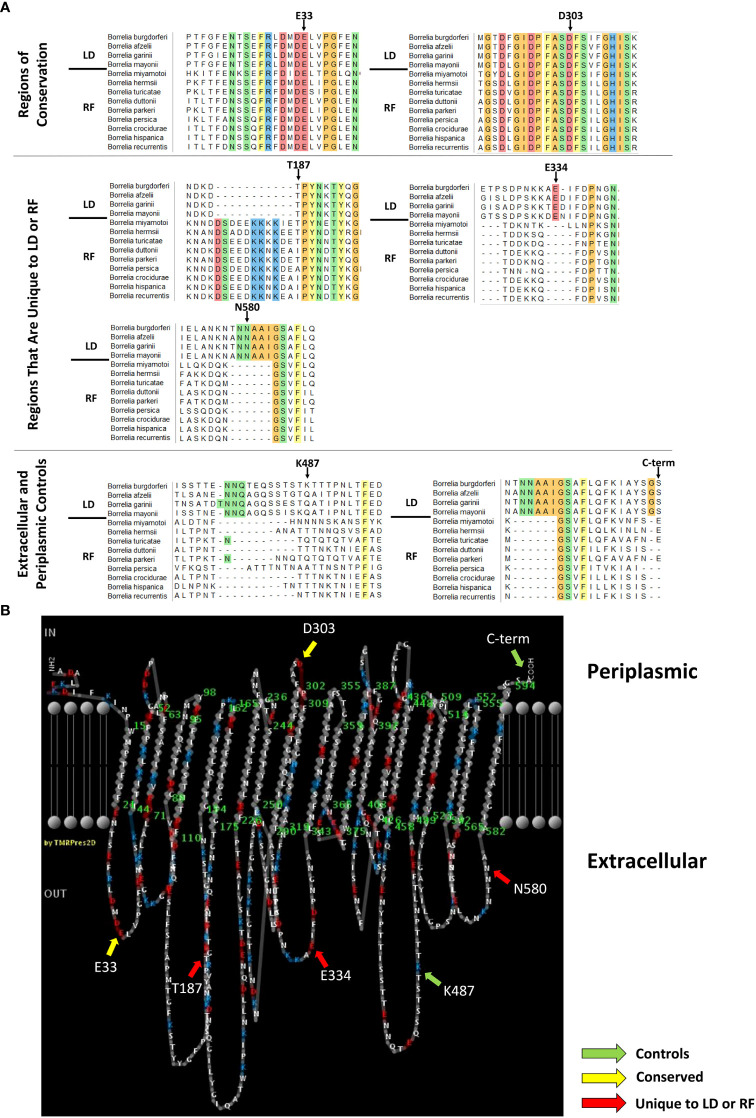
P66 domains selected for c-Myc tag insertion. **(A)** P66 sequences from Lyme disease (LD)- and relapsing fever (RF)-causing *Borrelia* species were aligned using Multiple Alignment Fast Fourier Transform. The first four sequences (above the black line) are P66 sequences from LD-causing *Borrelia*. The last nine sequences (below the black line) are P66 sequences from RF-causing *Borrelia*. Amino acids that are conserved in all species listed are highlighted and colored by side chain chemistry: yellow for aromatic, red for acidic, blue for basic, orange for nonpolar, and green for polar. Arrows indicate the amino acid for which the region is named and which is directly N-terminal to the c-Myc insertion. **(B)** PRED-TMBB prediction (Posterior Decoding) of the predicted periplasmic, transmembrane, and extracellular domains of LD-causing *B*. *burgdorferi* P66 with domains from **(A)** indicated with colored arrows.

c-Myc tags were introduced immediately C-terminally to each of the indicated amino acid residues shown in **Figure 1**. Outlined below is the method used to clone the c-Myc tag at the C-terminus of P66 (complemented on a plasmid; B31 A3-C-term^cp^). A c-Myc tag (EQKLISEEDL) was cloned into the C-terminus of *p66* on the pBSV2G vector (Elias et al., 2003) using Gibson Assembly. PCR was first performed on the pBSV2G + *p66* vector (Ristow et al., 2012) using primers *pBSV2G.obb0602u* (primer sequences found in **Supplementary Table S3**) and *Over C-term 1*. *A* second PCR product was amplified from the same vector using primers *pBSV2G.obb0604m* and *Over C-term 2*. Then pBSV2G was cut with SacI and SphI, combined with both PCR products and Gibson Assembly Master Mix (New England BioLabs, Ipswich, MA, USA), and incubated at 50°C overnight. The resulting product (3 μl) was electroporated into *E. coli* Top10 cells at 1800 V. Cells were plated on LB + 0.2% dextrose + gentamicin (5 μg ml^-1^). Plasmids were isolated from single, re-streaked *E. coli* colonies and were sequenced at MCLAB (South San Francisco, CA, USA) using primers described in **Supplementary Table S4**. Plasmids containing the correct sequence were methylated *in vitro* (Chen et al., 2008) and electroporated into *Borrelia burgdorferi* B31 A3 KO4 C3-14 (Δ*p66*) (Ristow et al., 2012) as previously described (Samuels, 1995). Potential *Borrelia burgdorferi* clones were selected for gentamicin resistance by growing in BSKII plates supplemented 40 μg ml^-1^ gentamicin and incubated at 33°C with 2% CO_2_ for two weeks. Single colonies were picked and grown in 5 ml BSKII + gentamicin (40 μg ml^-1^) at 33°C. After cultures reached a density >1 x 10^7^ cells ml^-1^, multiplex PCR (Bunikis et al., 2011) was performed to determine retention of genomic plasmids present in the parental strain, and production of c-Myc tagged P66 was determined through western blot analysis using rabbit polyclonal anti-c-Myc (Sigma, 1:10,000 dilution). This method was used to generate all the plasmid complemented c-Myc tagged P66 mutants in the B31 A3 background. Following successful plasmid construction in *E. coli*, the same vectors were transformed into the B313-WT and HB19-Δ*p66* backgrounds. Limiting dilution was performed to isolate clones for some of the HB19 mutants (Yang et al., 2004; Battisti et al., 2008).

Different plasmid constructs were necessary for chromosomal complementation (*p66^cc^*) of the c-Myc mutants in the B31 A3 background, which restore mutated *p66* alleles to the native locus on the chromosome. In brief, the constructs used for plasmid complementation were double-digested with SacI and SphI and ligated to pGEMT Easy (Promega, Madison, WI, USA) which was also double digested with SacI and SphI. Ligations were transformed into chemically competent XL-Gold *E. coli* and selected on LB agarose plates containing ampicillin. This resulted in Intermediate 1. PCR-amplification of a gentamicin resistance cassette with flanking MfeI restriction sites was performed. This PCR product and Intermediate 1 plasmid were both digested with MfeI, ligated together, transformed into chemically competent cells, and selected on agarose plates containing ampicillin and gentamicin. This yielded Intermediate 2. Finally, Intermediate 2 was digested with BspHI to excise the AmpR cassette. The digest was diluted in water and transformed into chemically competent *E. coli* cells. This yielded the final construct for transformation into B31 A3 Δ*p66*. Sequence confirmation was performed on every intermediate and final construct. Transformation into *Borrelia burgdorferi* Δ*p66* was performed as described for the c-Myc P66^cp^ strains except the methylation step was replaced with an overnight restriction digest with ApaI. All enzymes were purchased from New England BioLabs and used according to manufacturer’s instructions (Ipswich, MA, USA).

### Proteinase K cleavage assay

This procedure was performed as described previously with some modifications (Ristow et al., 2015). *Borrelia burgdorferi* strains were grown to > 3 x 10^7^ cells ml^-1^ in BSKII at 33°C. For each strain, cells were pelleted at 1,509 x *g* for 30 minutes at room temperature, then washed and concentrated to 1.6 x 10^8^ cells ml^-1^ in phosphate-buffered saline (PBS; 150 mM NaCl, 17 mM K_2_HPO_4_, 5 mM KH_2_PO_4_; pH 7.4). Cells were separated into 4 x 250 μl aliquots (4 x 10^7^ cells), pelleted at 11,200 x *g* for 8 minutes, resuspended in 0, 1, 10, or 50 μg ml^-1^ Proteinase K (Promega) in PBS, and incubated at 33°C for 1 hour. After incubation, phenylmethylsulfonyl fluoride (Thermo Fisher Scientific, NJ, USA) was added to a final concentration of 0.4 mM and incubated for 15 minutes at room temperature. Cells were pelleted at 11,200 x *g* for 8 minutes and resuspended in PBS to a density of 2 x 10^9^ cells ml^-1^. An equal volume of 2x sample buffer (made of 20 ml 0.5 M Tris + 20 ml 10% SDS + 60 ml saturated sucrose + 1 ml 10% NaN_3_ + bromophenol blue to adjust color) + 2% β-mercaptoethanol was added to each sample before boiling for 5 minutes at 100°C. Proteins were separated by running 2.5 μl (2.5 x 10^6^ cells) on SDS-PAGE (10% polyacrylamide), transferred to a PVDF membrane, and probed with mouse anti-flagellin (monoclonal antibody H9724 (Barbour et al., 1986) at 1:500 dilution) and either rabbit anti-P66 or rabbit anti-c-Myc (Sigma-Aldrich, St. Louis, MO, USA; at 1:10,000 dilution) primary antibodies. Anti-rabbit-HRP conjugated and anti-mouse-HRP conjugated antibodies (both at 1:10,000 dilutions; Promega) were used as secondary antibodies. Bands were visualized using ECL substrate (Bio-Rad, Hercules, CA, USA) and chemiluminescence on a Bio-Rad Chemidoc. Imagelab (5.2.1) was used to quantify the density of each flagellin band relative to the 0 μg ml^-1^ Proteinase K flagellin band for each *Borrelia burgdorferi* strain. Similarly, the density of the P66 (or c-Myc) band was quantified relative to the 0 μg ml^-1^ Proteinase K concentration P66 (or c-Myc) band for each *Borrelia burgdorferi* strain. The percent decrease of P66 (or c-Myc) was calculated by subtracting the relative P66 (or c-Myc) band density from the relative flagellin band density, then dividing by the relative flagellin band density.


$$\left(\frac{\left(relativeflagellindensity-relativeP66(orc-Myc)density\right)}{relativeflagellindensity}\right)\times 100$$

### Immunofluorescence microscopy of c-Myc epitope-tagged P66 mutants

Immunofluorescence microscopy was performed as outlined previously with modifications (Kenedy et al., 2014). *Borrelia burgdorferi* cultures were grown to 3 x 10^7^ – 2 x 10^8^ cells ml^-1^ in BSKII at 33°C. For each strain, cells were pelleted at 11,200 x *g* for 8 minutes at room temperature and resuspended in PBS + 0.1% BSA at a density of 4 x 10^7^ cells ml^-1^. Cells were split into 2 x 250 μl aliquots (for live cell staining and permeabilized cell staining) and pelleted at 11,200 x *g* for 8 minutes at room temperature. For live cell staining, cells were blocked in 3% BSA (in PBS) for 30 minutes at room temperature, pelleted at 11,200 x *g* for 8 minutes, and resuspended in 3% BSA with rabbit anti-c-Myc (Sigma-Aldrich, St. Louis, MO, USA; at 1:1000 dilution) and mouse anti-flagellin (monoclonal antibody H9724 at 1:1000 dilution) antibodies for 1 hour at room temperature. Cells were pelleted at 11,200 x *g* for 8 minutes, and washed 3x in PBS + 0.1% BSA. After the final centrifugation, cells were resuspended in 100 μl of PBS, spotted (20 μl) on a glass microscope slide, incubated on the slide for 20 minutes, fixed with ice-cold acetone for 10 minutes, and incubated with AF-488 goat anti-rabbit and AF-568 goat anti-mouse antibodies (Life Technologies, Carlsbad, CA, USA; each at 1:1000 in 3% BSA) for 1 hour at room temperature. Cells were washed 3x with 3% BSA, incubated with 10 μg ml^-1^ DAPI (Invitrogen) for 30 minutes at room temperature, washed 3x (first wash: 3% BSA, second wash: 0.1% BSA, third wash: PBS) and mounted in 10 μl of ProLong Diamond Antifade Mountant (Invitrogen, Waltham, MA, USA). The staining of permeabilized cells was performed slightly differently. After splitting the cells as described above, cells were washed two more times in PBS + 0.1% BSA, resuspended in 100 μl PBS, spotted on microscope slide, incubated for 20 minutes at room temperature, fixed with ice-cold acetone for 10 minutes, and blocked with 3% BSA. Cells were then incubated with rabbit anti-c-Myc (1:1000) and mouse anti-flagellin (1:1000) antibodies for 1 hour at room temperature. Secondary antibody incubations, DAPI incubations, and mounting procedures were carried out as described for live cell staining. Immunofluorescence microscopy images were taken at 100x objective under oil immersion using a Nikon Eclipse 80i. Data are representative of at least three independent experiments.

### Non-ionic detergent extraction

*Borrelia burgdorferi* strains were grown to >5 x 10^7^ cells ml^-1^. Cells (2 x 10^8^) were pelleted at 1,509 x *g* for 30 minutes at room temperature, washed in 0.1% BSA in PBS, and pelleted again at 11,200 x *g* for 8 minutes. Cells were resuspended in HEPES buffered saline (HBS; 25 mM HEPES, 150 mM NaCl, 0.1% sodium azide, 1 mM benzamidine, 0.01 TIU ml^-1^ aprotinin; pH 7.8), pelleted at 11,200 x g for 8 minutes, resuspended in 100 µl HBS, and 20 µl was kept as the total cell (TC) sample. The remaining cells were pelleted as before, resuspended in 80 µl HBS + 25 mM octylglucoside, and incubated at 4°C with rocking for 2 hours. Supernatants (soluble material) and pellets (insoluble material) were separated by centrifugation at 16,000 x *g* for 10 minutes at 4°C. The supernatant was re-centrifuged to remove any remaining insoluble material. The pellet was washed by resuspending in 80 µl HBS + 25 mM octylglucoside, re-centrifugation at 16,000 x *g*, and removal of the supernatant. SDS-PAGE and western blotting were performed as described in the Proteinase K methods. The rat polyclonal anti-Bb0405 antibodies and anti-OppAIV antibodies were generously provided by Dr. Darrin Akins and used at 1:1000 and 1:5000 respectively.

### Vancomycin susceptibility assay

*Borrelia burgdorferi* B31 A3 and derivative strains were grown to >5 x 10^7^ cells ml^-1^ in 5 ml BSKII + appropriate selection antibiotics (200 µg ml^-1^ kanamycin for Δ*p66* and 200 µg ml^-1^ kanamycin + 40 µg ml^-1^ gentamycin for all Δ*p66* derived strains) at 33°C. Cells were diluted into 5 ml BSKII with selection antibiotics and ± vancomycin (1 μg ml^-1^) at a starting density of 1 x 10^6^ cells ml^-1^. Cultures were counted daily (including the day 0 time point) using a Petroff-Hausser counting chamber under dark-field microscopy. All intact *Borrelia burgdorferi* cells were counted, but assessment of subjective motility was not performed for each individual cell. Data represent three independent experiments for each c-Myc P66^cp^ strain and six independent experiments for the controls. Data were analyzed using linear regression (GraphPad Prism 7.02) to determine whether the growth rates of *Borrelia burgdorferi* B31 A3 and all derivative strains were different in the presence and/or absence of vancomycin. The same method was used for the B31 A3 c-Myc P66^cc^ strains.

### Integrin-binding assay

Purified integrin a_v_β_3_ [extracellular domains only (Zhu et al., 2010)] was diluted in cold HBSC (25 mM HEPES pH 7.8, 150 mM NaCl, 2 mM MnCl_2_, 0.2 mM CaCl_2_) to 50 nM. 96-well plates (Costar catalog number 3590) were pre-chilled and 50 µl of 50 nM integrin or 50 µl of the HBSC buffer control were added to each well. The plates were incubated overnight at 4°C to immobilize the integrin.

*Borrelia burgdorferi* strains in the HB19 background were grown in the presence of appropriate antibiotics in MKP. Culture density was monitored by darkfield microscopy until they reached exponential phase. Bacterial cells were pelleted at 4,300 x g for 10 min at room temperature. The cells were washed twice in 500 µl of HBSC + 0.2% BSA + 0.04% dextrose and centrifuged at 11,360 x g for 8 min at room temperature. The cells were ultimately resuspended in 600 µl of HBSC + 1% BSA + 0.2% dextrose (resuspension buffer). Cells were diluted to 5x10^7^ cells ml^-1^ in resuspension buffer. One hundred microliters of each cell suspension were set aside for DNA extraction and qPCR as described below.

While the cells were being prepared, the 96-well plate with integrin was washed 3 times with 200 µl per well of ambient temperature HBSC, then blocked for 1 hr with resuspension buffer at ambient temperature. When the hour had elapsed and the diluted cells were prepared, the blocking buffer was removed and replaced with 50 µl of diluted cells or resuspension buffer control. A total of 4 technical replicate wells were inoculated per condition. The inoculated plate was centrifuged at 670 x g for 20 min at room temperature to facilitate an interaction between the integrins and the bacteria, then incubated at room temperature for 1 hr with rocking. The inoculum was removed and the plate was washed 3 times with 200 µl per well of room temperature HBSC, emptied, and immediately processed for DNA extraction.

Bacterial DNA was harvested similarly to that previously described, except 100 ul of inoculum was analyzed instead of 50 ul (Surdel et al., 2022). The sets of four technical replicate wells were combined such that two wells were pooled into a single DNA collection tube, for a total of 2 tubes per condition. Purification of sample and inoculum DNA proceeded according to manufacturer’s instructions (DNeasy Blood & Tissue Kit; Qiagen, Hilden, Germany).

Quantitative PCR was performed using PR1MA qMAX Green qPCR Mix (MidSci, St. Louis, MO, USA, PR2000-N). Each DNA sample was quantified in triplicate on a BioRad CFX96 cycler using primers targeting *recA* that were previously described (Morrison et al., 1999). Standard curves were created using genomic DNA isolated from *Borrelia burgdorferi* isolate HB19, converting the mass of DNA in each standard to genomes per reaction using the *Borrelia burgdorferi* genome size. Samples from the adhesion assay were quantified and fit to the standard curve in BioRad CFX Manager to determine starting quantities in each reaction. Data were exported to Microsoft Excel for further analysis.

Preliminary data demonstrated differences in percent bound for varied inoculum concentrations (data not shown). Therefore, experiments with >3-fold intra-experimental differences or >10-fold inter-experimental differences in the bacterial inoculum concentration were excluded from analysis. All technical replicates for each day were averaged, and data from three replicate experiments were combined for analysis. All data were graphed and statistical analyses were performed in GraphPad Prism 9.3.1. Statistical significance to a selected control strain was tested by one-way ANOVA with Dunnett correction for multiple comparisons.

### Isolation of outer membrane proteins

The isolation of the B-fraction (membrane fraction with an enrichment of outer membrane proteins) of the different *Borrelia* strains was performed as described previously (Magnarelli et al., 1989). B-fractions were solubilized in ultrapure water and protein concentrations were determined using the Qubit™ Protein assay kit (Invitrogen). Samples were stored at -20˚C until used for SDS-PAGE, Blue Native-PAGE, immunoblotting, and black lipid bilayer assays. Notably, the B-fraction does not exclusively contain P66 protein, but P66 is easily identified from other porins by its high channel conductance.

### SDS-PAGE of B-fractions

Approximately 10 µg of B-fraction was mixed with NuPAGE LDS Sample buffer (4X) (Invitrogen) and 2.5% (v/v) β-mercaptoethanol, then boiled at 100°C for 5 min. Ten microliters of each sample was loaded into NuPAGE™ Novex 4-12% Bis-Tris gels (Invitrogen) and electrophoresis was performed according to manufacturer’s instructions. The Precision Plus Protein™ Kaleidoscope™ Prestained protein Standards (Bio-Rad) were used as molecular mass markers.

### Blue native-PAGE (BN-PAGE) of B-fractions

Approximately 10 µg of B-fraction was solubilized in 30 µl of 1.25% digitonin (Invitrogen) by incubation for 15 min at room temperature with intermittent vortexing. After solubilization, the samples were centrifuged at 16,000 x *g* for 20 min at 20˚C to pellet the insoluble material. The supernatant (20.6 µl) was mixed with Native PAGE 4X Sample Buffer (7.5 µl) (Invitrogen) and 5% (w/v) Coomassie blue G-250 (1.86 μl) (Invitrogen) just prior to loading samples onto the Blue Native gel.

NativePAGE™ Novex^®^ 4-16% Bis-Tris gels were used according to the manufacturer´s instructions (Invitrogen). The first third of the run was performed with Dark Blue Cathode Buffer (Invitrogen), and the remaining two-thirds were performed with Light Blue cathode Buffer (Invitrogen). The Native Mark Unstained (Invitrogen) was used as the molecular mass standard.

### Western blots of B-fractions

SDS-PAGE and BN-PAGE gels were blotted onto BioTrace™ PVDF membranes (Merck Millipore, Carrigtwohill, Ireland). Electrophoretic transfer of proteins was performed for 90 min at 30 V, 170 mA in the case of SDS-PAGE gels, or for 60 min at 25 V, 130 mA in the case of BN-PAGE gels. After transferring, in the case of BN-PAGE, the membranes were fixed in 8% acetic acid solution for 15 min at room temperature and rinsed with deionized water. Membranes were blocked with 5% milk in PBS overnight at room temperature, then rinsed with 0.05% Tween in PBS. Membranes were subjected to immunoblotting using either a rabbit polyclonal antiserum raised against P66 (D8713, diluted to 1:10,000), or a polyclonal rabbit anti-c-Myc antibody (C3956, Sigma, Saint Louis, MO, USA) diluted to 1:10,000 (for SDS-PAGE blots) or to 1:500 (for BN-PAGE blots) in 2.5% milk in PBS. Membranes were then washed three times for 5 min each with 0.05% Tween in PBS and incubated with an anti-rabbit secondary antibody coupled to horseradish peroxidase (ECL™ anti-rabbit IgG, Horseradish peroxidase linked whole antibody, Sigma). Membranes were washed three times for 5 min each with 0.05% Tween in PBS and bound antibodies were detected using Amersham ECL™ Prime Western Blotting detection reagents (GE Healthcare, Buckinghamshire, UK).

### Black (planar) lipid bilayer assays

The method for black lipid bilayer experiments has been described previously (Benz et al., 1978). The bilayer equipment consisted of a Teflon chamber with two compartments separated by a thin wall and connected by a small circular hole with an area of about 0.4 mm^2^. The two compartments were filled with 1 M KCl salt solution and the membranes were formed by painting a 1% (w/v) solution of 1,2-diphytanoyl-sn-glycero-3-phosphocholine (DPhPC) (Avanti Polar Lipids, Alabaster, AL, USA) in n-decane over the hole. The B-fractions were prepared at a concentration of 0.1 µg µl^-1^, then diluted 1:10 in 1% Genapol X-080 (Fluka, Spain) and added to the aqueous phase on both sides of the chamber after the membrane had turned black. The membrane current was measured with a pair of Ag/AgCl electrodes with salt bridges switched in series with a voltage source and a highly sensitive current amplifier (Keithley 427, Lower Lake, CA, USA). The voltage applied was 20 mV. The signal was monitored with a strip chart recorder (Rikadenki, Tokyo, Japan). Experiments were performed in duplicate (unless otherwise stated) at room temperature, using a single B-fraction from each strain.

### Mouse infections

Animals were housed according to institutional guidelines and fed and watered *ad libitum*. The Medical College of Wisconsin Institutional Animal Care and Use Committee approved all work with animals.

### Short-term infection model

Female C3H/HeN mice (6-8 weeks old; Charles River Laboratories, Wilmington, MA, USA) were infected as previously described but with some differences (Caine and Coburn, 2015). Strains of interest were cultured to exponential phase in BSKII medium and profiled for genomic plasmid content by multiplex PCR prior to infections (Bunikis et al., 2011). Culture density was quantified by darkfield microscopy and cells diluted to 1x10^9^ cells ml^-1^ in PBS + 0.2% normal mouse serum (from C3H/HeN mice). A volume of this inoculum was also set aside for quantification by qPCR. Prior to infection, mice were weighed and anesthetized with a cocktail of ketamine at 100 mg kg^-1^ and xylazine at 10 mg kg^-1^ with a 27 G needle. Once anesthetized, the tail was warmed under a heat lamp, sterilized with 70% ethanol, and the mouse was injected with 100 µl of inoculum through the tail vein. The mouse was kept on a warming pad for one hour; a second dose of anesthetic was administered 40 minutes into the infection. At the conclusion of the hour, the chest area was sterilized with 70% ethanol and blood obtained by cardiac puncture into a syringe with Ware’s solution and placed on ice. The chest cavity was opened and an incision was made in the right atrium of the heart allowing drainage. A bag of saline was attached to a 21 G x 3/4-winged infusion kit which was inserted into the left side of the heart. Saline was perfused at 1 ml min^-1^ for 6 mins and outflow collected with an absorbent pad and gauze. Following perfusion, lung, heart, liver, spleen, and bladder tissues were collected and rinsed with PBS. In addition, the skin of the left hind leg was then stripped away, and the foot cut off to allow collection of the ankle joint. The right ear was also collected and rinsed with PBS. All samples were placed on dry ice. Blood samples were spun at 16,100 x g for 15 mins at 5°C to separate out the serum, which was discarded. Tissues and blood pellets were stored at -80°C until further DNA processing by DNeasy blood and tissue kit (Qiagen) according to manufacturer’s instructions.

qPCR reactions were performed in triplicate using 50 ng of DNA per reaction on a CFX connect real time system. *Borrelia burgdorferi* genomes were amplified using Qiagen QuantiFast SYBR green master mix at 95.0°C for 5 min, then 40 cycles of 95.0°C for 10 s and 58.6°C for 30 s, 95°C for 1 min, and 50°C for 1 min. Primers were directed against the *recA* gene as previously described (Morrison et al., 1999). Mouse genomes in each DNA sample were also amplified using the same cycling conditions but with primers designed to amplify mouse β-actin gene (Ristow et al., 2015). Genomes were quantified using a standard curve generated from purified *Borrelia burgdorferi* B31 A3 genomic DNA (gDNA) or C3H/HeN mouse liver gDNA. Mouse gDNA isolated from liver tissue of C3H/HeN mice was added to each bacterial DNA standard at a concentration of 20 ng µl^-1^ to mimic the mouse gDNA present in each tissue DNA sample. *Borrelia burgdorferi* genomes were normalized to the inoculum, as determined by qPCR of an inoculum sample, and normalized to the total amount of input DNA in the reaction. Medians and ranges were plotted for each data set and analyzed for statistical significance by the Kruskal-Wallis test and corrected for false discovery by the two-stage step-up method of Benjamini, Krieger, and Yekutieli using GraphPad Prism.

### Long-term infections

To test infectivity as determined by dissemination from a skin inoculation site that better mimics natural infection, female C3H/HeN mice (6-8 weeks old; Charles River Laboratories) were inoculated subcutaneously with 1x10^5^ cells of exponential phase B31 A3 WT, Δ*p66*, and B31 A3-P66^cc^ mutants. Two clones of each c-Myc mutant were tested in groups of 5 mice (i.e. 10 mice per c-Myc mutant). Immediately prior to inoculation, all strains were verified to contain the genomic plasmid content of the parental B31 A3 by multiplex PCR and to produce P66 protein as evidenced by western blot. Mice were euthanized by CO_2_ inhalation at 28 days post-inoculation. Multiple tissues were collected and used to inoculate 5 ml BSKII cultures: blood, bladder, heart, knee, ankle, inoculation site skin, and ear. Cultures were kept for up to 8 weeks and monitored for spirochete growth by darkfield microscopy. Unfortunately, an incubator malfunction prevented monitoring of the B31 A3-T187^cc^ and B31 A3-K487^cc^ cultures past the first week. However, the data demonstrated 6/10 positive mice for the B31 A3-T187^cc^ infections and 10/10 positive mice for the B31 A3-K487^cc^ infections.

### ID_50_ determinations

Infections for ID_50_ determination were set up identically to the long-term infections except that doses of 1x10^3^, 1x10^5^, 1x10^7^, and 1x10^9^ spirochetes per mouse were inoculated. Only the c-Myc E33^cc^ and c-Myc D303^cc^ mutants were tested. Cultures were monitored for spirochete growth by darkfield microscopy for 4 weeks. ID_50_ values were calculated as previously described (Reed and Muench, 1938).

## Results

### *In silico* analyses of P66 were used for directed mutagenesis and topological predictions

The P66 amino acid sequences from LD-causing *Borrelia* and RF-causing *Borrelia* were aligned using Multiple Alignment Fast Fourier Transform (MAFFT). MAFFT was chosen to align the sequences because it has a post-processing step that may correct misalignments, and it was shown to be more accurate and consistent than other multiple sequence alignment programs (Katoh et al., 2002; Pais et al., 2014). LD-causing *Borrelia* used in the alignment were *Borrelia burgdorferi* B31 (NP_212737), *B. afzelii* Pko (YP_710051), *B. garinii* Pbi (YP_073044), and *B. mayonii* (WP_075552264). RF-causing *Borrelia* used in the alignment were *B. miyamotoi* LB-2001 (AGT27550.1), *B. hermsii* MTW (AHH14103.1), *B. turicatae* 91E135 (AAX17926.1), *B. parkeri* SLO (AHH09322.1), *B. persica* (WP_024653437.1), *B. duttonii* Ly (ACH93541.1), *B. crocidurae* str. Achema (AFI31391.1), *B. hispanica* (WP_024654868.1), and *B. recurrentis* A1 (ACH94835.1).

The MAFFT alignment revealed twelve regions that are highly conserved between all *Borrelia* species examined, two of which are shown (**Figure 1A**). The alignment also revealed several regions of variability. There were three sites (i.e. E334, K487, and N580) that contained insertions of amino acids in the LD-causing *Borrelia* sequences as compared to the RF-causing *Borrelia* sequences. The opposite was also found. There was one site (i.e. T187) that contained an insertion of amino acids in the RF-causing *Borrelia* sequences that were absent in the LD-causing *Borrelia* sequences. The amino acid insertion in the RF-causing *Borrelia* sequences was adjacent to one of the two critical Asp residues identified as the integrin binding domain of *Borrelia burgdorferi* P66 (Defoe and Coburn, 2001). The lysine at residue 487 is a surface-exposed trypsin cleavage site on *Borrelia burgdorferi* P66 (Bunikis et al., 1998; Kenedy et al., 2014). The other regions have not been characterized previously.

It has been shown that amino acid insertions/deletions are more likely to occur on non-structured loops than in transmembrane regions (Pascarella and Argos, 1992). Therefore, we investigated if the amino acid insertions/deletions among the different species of *Borrelia* were located on regions that were predicted to be transmembrane, periplasmic, or extracellular. We used a web-based server called PRED-TMBB that uses a Hidden Markov Model to discriminate β-barrel proteins and predicts transmembrane, periplasmic, and extracellular residues based on three algorithms (i.e. Viterbi, N-best, and Posterior Decoding (Bagos et al., 2004b)) to predict where the amino acid insertions/deletions are located on P66 from B. *burgdorferi.* Although Kenedy *et al.*, published PRED-TMBB results of P66 in 2014 (Kenedy et al., 2014), we repeated the prediction because the server is updated on a regular basis when new crystal structures become available (Bagos et al., 2004b).

P66 from *Borrelia burgdorferi* B31 was submitted to PRED-TMBB webserver. The server produces a score for each submitted protein. If the score is below the predefined threshold of 2.965, then the protein is considered a β-barrel protein. The model gave *Borrelia burgdorferi* P66 a score of 2.882 indicating that it predicts P66 to be a β-barrel protein (Bagos et al., 2004b). Viterbi and N-best algorithms predicted P66 to have 24 transmembrane domains and 12 extracellular loops (not shown). The Posterior Decoding algorithm predicted P66 to have 22 transmembrane domains and 11 extracellular loops (**Figure 1B**). Some of the conserved regions span predicted periplasmic loops plus contiguous transmembrane domains, others span extracellular loops plus contiguous transmembrane domains, and still others are exclusively in predicted extracellular loops. All of the amino acid insertions/deletions identified are predicted to be on surface-exposed loops of *Borrelia burgdorferi* P66 except for D303 and C-term.

To investigate whether the selected domains are exposed on the surface of *Borrelia burgdorferi* and/or are critical for P66 localization or function, we inserted c-Myc (EQKLISEEDL) epitope tags on the C-terminal side of T187 (mature protein sequence), E334, K487 (as described in (Kenedy et al., 2014)), and N580 in *Borrelia burgdorferi* P66 to correspond with unique domains between LD and RF *Borrelia*. c-Myc epitope tags were also inserted on the C-terminal side of E33 and D303, both of which lie within conserved domains. E33 is in a predicted extracellular domain and D303 is in a domain predicted to be periplasmic (**Figure 1B**).

The c-Myc tagged P66 proteins were expressed from the pBSV2G vector (Elias et al., 2003) in three different strains of *Borrelia burgdorferi:* B31 A3 KO4 C3-14, B313, and HB19 KO4 C3-14. B31 A3 KO4 C3-14 (abbreviated as ‘Δ*p66’* throughout this paper) is a derivative of the infectious B31 A3 strain. The KO4 strains (Δ*p66*) have a kanamycin resistance cassette replacing approximately the middle third of the *p66* gene (Ristow et al., 2012). We have chosen to maintain the Δ*p66* nomenclature because there is no detectable protein produced, and for consistency with our previously published work (Coburn and Cugini, 2003; Ristow et al., 2012). The B31 A3 Δ*p66* derivative strains, which have c-Myc-tagged *p66* complemented on a plasmid (c-Myc P66^cp^), were used to determine if the c-Myc insertion disrupted P66 localization to the outer membrane and porin function. The advantage of this strain background is that it does not produce endogenous P66, so P66 is overproduced from the plasmid and exclusively tagged with c-Myc. Notably, restoration of the WT *p66* allele on the pBSV2G vector does not restore infectivity, likely due to the overproduction of the protein (Ristow et al., 2012). Therefore, for infection studies, the c-Myc P66-encoding genes were restored to the native locus on the chromosome in B31 A3 Δ*p66* through homologous recombination. This method of complementation has been shown previously to restore infectivity levels back to WT levels (Ristow et al., 2012).

### P66 localization is altered in the N580 mutant, but not the other c-Myc P66 mutants

**Figure 2:**
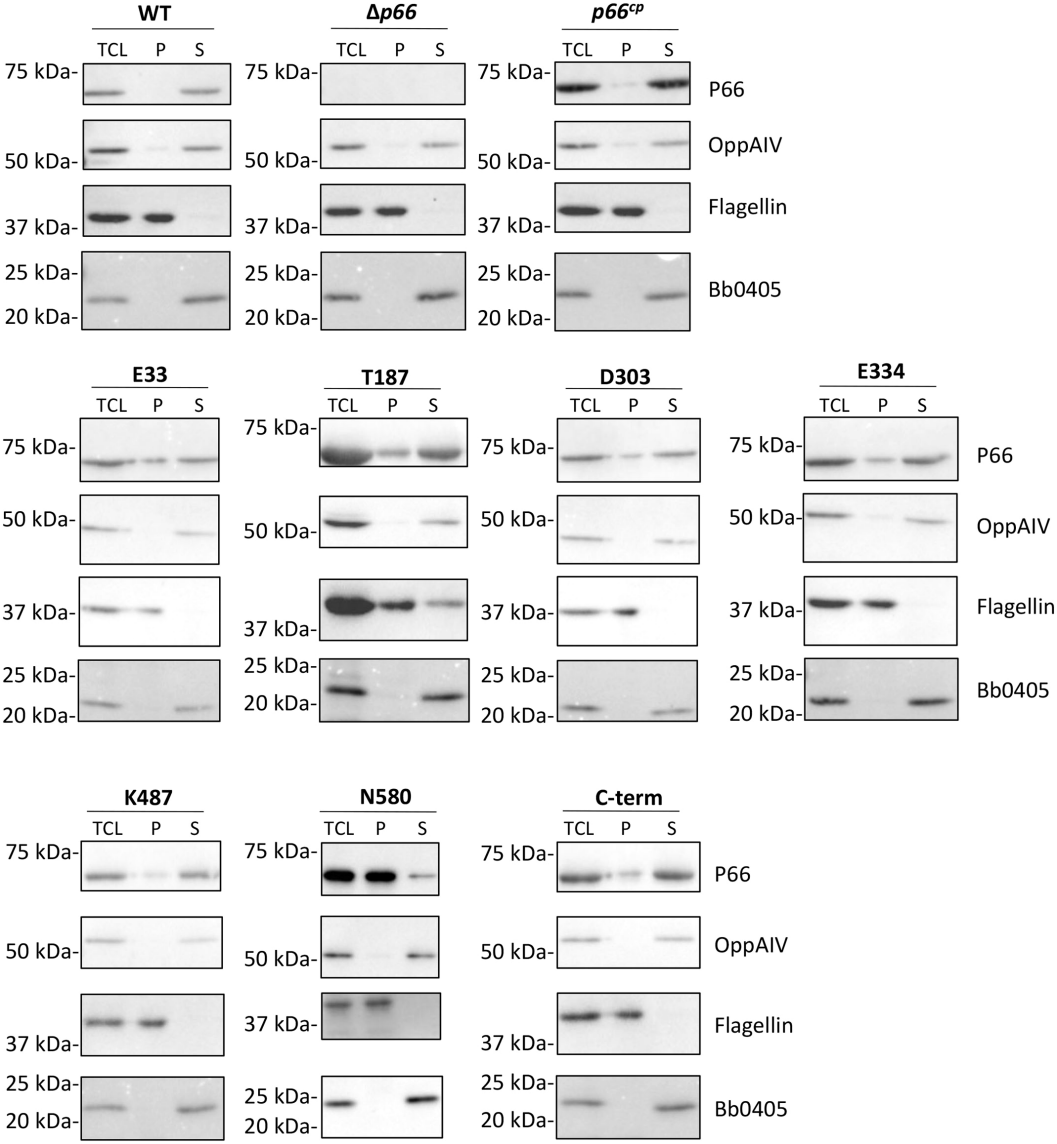
Non-ionic detergent extraction of P66. B31 A3 WT, Δ*p66*, and derivative strains (B31 A3 c-Myc P66^cp^) were treated with HEPES buffered saline and octyl β-D-glucopyranoside at 4°C for 2 hours. Total cell lysate (TCL) was centrifuged to separate pellet (P) and supernatant (S) fractions. Fractions were subjected to SDS-PAGE and western blotting for the indicated proteins. Representative images are shown.

In order to determine whether the c-Myc epitope tags at the various locations affected solubilization of P66 in a non-ionic detergent, an octylglucoside extraction was performed (**Figure 2**). All of the detectable P66 protein from WT B31 A3 was localized in the supernatant (soluble: S) fraction. The majority of WT P66 overproduced from the pBSV2G plasmid in the Δ*p66* background (*p66^cp^*) was also localized in the supernatant fraction, but a small portion of P66 remained in the pellet (insoluble; P) fraction (Ristow et al., 2012). The inner membrane protein OppAIV and the outer membrane protein Bb0405 were both localized in the supernatant fraction, while flagellin remained in the pellet fraction. The majority of the c-Myc tagged P66 proteins were localized in the supernatant fraction. The only exception was P66 with the c-Myc insertion at N580, for which the majority of P66 remained in the pellet fraction.

Proteinase K cleavage assays were performed to determine if the c-Myc tagged P66 proteins were exposed on the surface of *Borrelia burgdorferi*. Proteinase K is a serine protease that cleaves the peptide bond adjacent to the carboxyl group of aliphatic and aromatic amino acids (Ebeling et al., 1974). Proteinase K does not penetrate the outer membrane of *Borrelia burgdorferi* (Probert et al., 1995). If the outer membrane of *Borrelia burgdorferi* is not disrupted, then the periplasmic flagellin (Charon et al., 2012) is not degraded upon incubation with Proteinase K, while outer membrane lipoproteins OspA, OspB, OspC, and OspD are degraded (Probert et al., 1995). Proteinase K cleavage assays are routinely used to demonstrate surface exposure of proteins in *Borrelia* (Barbour et al., 1984; Bunikis et al., 1995; Bunikis et al., 1996; Kenedy et al., 2014; Ristow et al., 2015).

**Figure 3:**
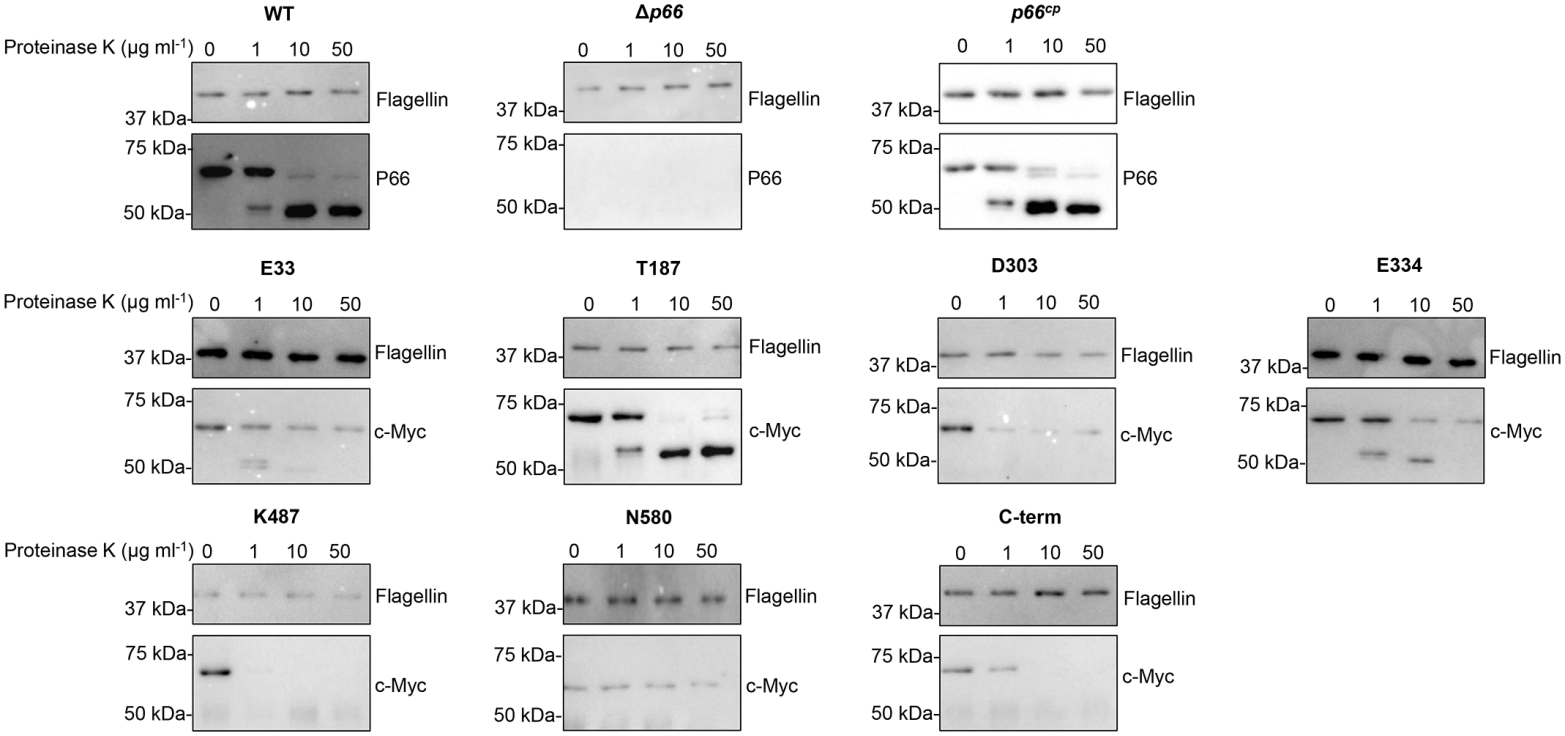
B31 A3 c-Myc P66^cp^ proteins are exposed on the surface on *B*. *burgdorferi*. Cells were incubated with increasing concentrations of Proteinase K for one hour at 33°C. Following deactivation of Proteinase K with PMSF, cell lysates were subjected to SDS-PAGE and western blotting. Blots were probed with antibodies to flagellin and P66 or c-Myc. Blots are representative of at least three independent assays.

In our studies, P66 produced by B31 A3 WT (parental strain of B31 A3 Δ*p66*) was degraded upon incubation with increasing concentrations of Proteinase K (0, 1, 10, 50 μg ml^-1^), while flagellin remained intact (**Figure 3**). At 10 µg ml^-1^ Proteinase K, 97% of P66 was cleaved in B31 A3 WT (**Supplementary Table S5**). This analysis was performed for all *Borrelia burgdorferi* strains tested. The degraded product at ~50 kDa has been observed previously and was interpreted to demonstrate that P66 is an integral outer membrane protein (Probert et al., 1995; Bunikis et al., 1996; Bunikis and Barbour, 1999; Ristow et al., 2015). Our Proteinase K results support previous conclusions that endogenous P66 is surface exposed. Incubation with 50 μg ml^-1^ Proteinase K did result in degradation of flagellin in some experiments making it difficult to accurately quantify the percent of P66 degraded compared to flagellin at that concentration. Therefore, conclusions were drawn from the samples incubated with 10 µg ml^-1^ Proteinase K concentration. B31 A3 Δ*p66* was included as a control for any potential non-specific antibody reactivity. Wild-type P66 expressed from the pBSV2G plasmid in Δ*p66* (*p66^cp^*) was degraded by Proteinase K, indicating that it is exposed on the surface of *Borrelia burgdorferi*. P66 proteins with c-Myc insertions at E33, T187, D303, E334, K487, and C-terminus were all degraded upon incubation with increasing concentrations of Proteinase K, while flagellin remained intact. At least 60% of c-Myc tagged P66 proteins produced in each strain were cleaved with 10 μg ml^-1^ of the enzyme (**Supplementary Table S5**). This demonstrates that the c-Myc insertion at these sites does not prevent surface localization of P66. The P66 protein with a c-Myc insertion at N580 exhibited aberrant localization as evidenced by its resistance to Proteinase K digestion. Interestingly, we did not observe the degraded product at ~50 kDa in the P66 proteins with c-Myc insertions at D303, K487, or C-terminus (**Figure 3**). This could be due to the c-Myc epitope being outside of the 50 kDa fragment, as we used a c-Myc antibody to probe the immunoblots. Another explanation is that the enhanced proteolysis observed in the mutant P66 forms is indicative of conformational changes.

### Immunofluorescence demonstrates that the E33, T187, E334, and K487 domains are extracellular while the C-terminus is not

In 2014, Kenedy *et al.* demonstrated through immunofluorescence microscopy that a c-Myc tag inserted at K487 was exposed on the surface of *Borrelia burgdorferi*, while a c-Myc tag inserted at the C-terminus of P66 was not detected on the surface of *Borrelia burgdorferi* (Kenedy et al., 2014). Therefore, we also used this method to determine whether c-Myc-tagged P66 domains were surface-exposed. The c-Myc-tagged K487 mutant was used as a positive control and the c-Myc-tagged C-terminus mutant was used as a negative control in our immunofluorescence microscopy studies. *Borrelia burgdorferi* strain B313, a non-infectious, high passage derivative of B31, was utilized for this experiment (Sadziene et al., 1993). One advantage of using this strain is that it does not produce OspA, OspB, OspC, or OspD (Sadziene et al., 1993). OspA and OspB have been previously reported to interact with P66 and shield epitopes from antibody binding during *in vitro* cultivation of *Borrelia burgdorferi* (Bunikis and Barbour, 1999). One disadvantage is that B313 produces endogenous P66, and multiple attempts to generate a P66 knockout in B313 by our laboratory failed.

Cells were subjected to two different staining procedures: live cell staining and permeabilized cell staining. For permeabilized cell staining conditions, acetone was used to permeabilize the cells prior to probing with anti-c-Myc and anti-flagellin antibodies. The permeabilized cell staining was used to determine whether all strains were producing c-Myc tagged P66 proteins. Under live cell staining conditions, cells were suspended in 3% BSA + anti-c-Myc and anti-flagellin antibodies prior to placement on microscope slides. The live cell staining was used to determine whether the c-Myc tag was surface exposed. Flagellin staining was used to determine whether the bacterial outer membrane was intact in the live cells, and to ensure that the antibody was reactive in the permeabilized cells.

**Figure 4:**
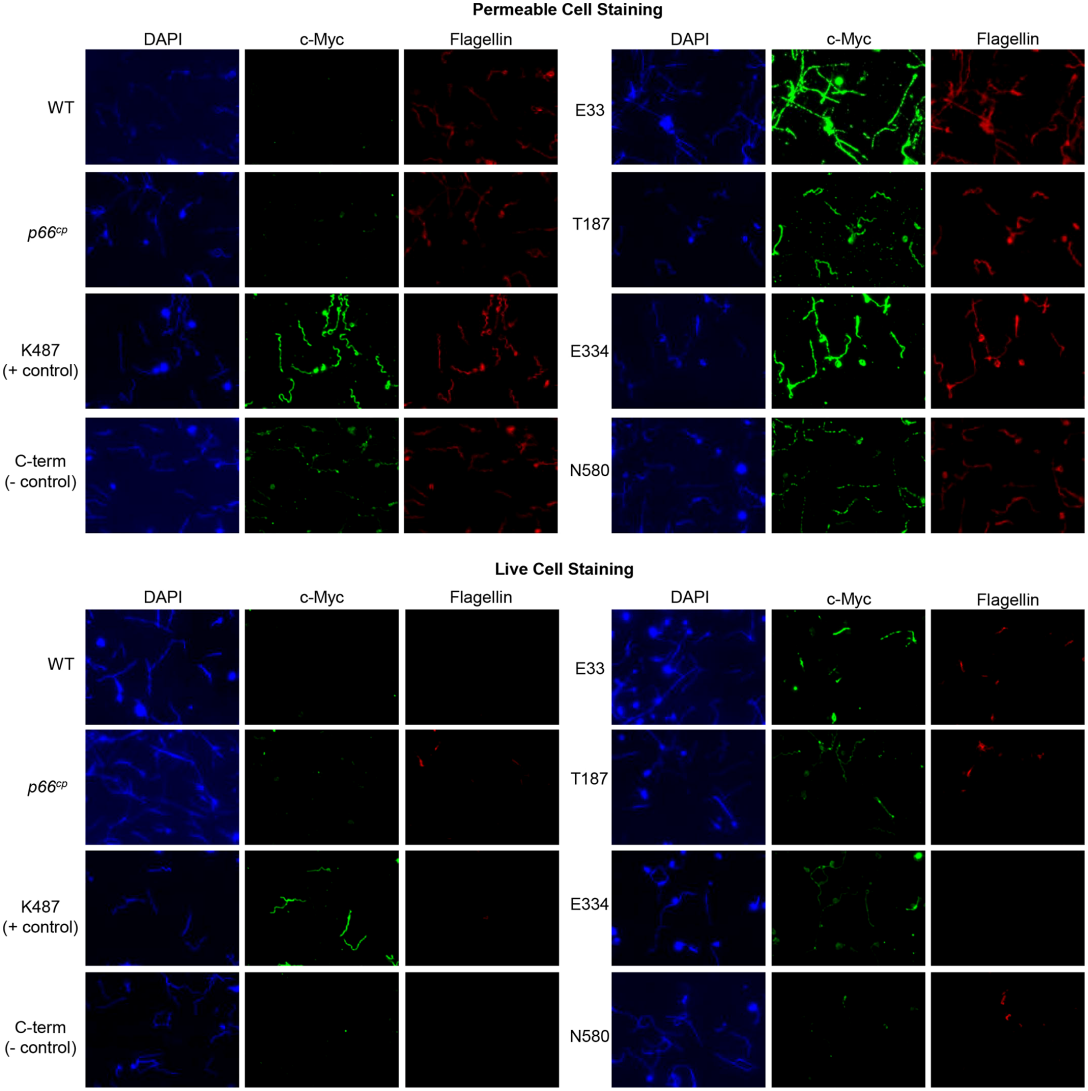
Immunofluorescence microscopy demonstrates surface localization of c-Myc-tagged P66 domains. Top: Permeable cell staining. B313 c-Myc P66^cp^ cells were permeabilized with ice-cold acetone, probed with anti-c-Myc and anti-flagellin antibodies, stained with DAPI, and mounted in Pro-Long Diamond mounting media. Bottom: Live cell staining. Cells were probed with anti-c-Myc and anti-flagellin antibodies, fixed with ice-cold acetone, stained with DAPI, and mounted in Pro-Long Diamond mounting media. Immunofluorescence microscopy images were taken at 100x oil immersion. Images are representative of at least 3 independent experiments.

As expected, B313 WT and B313 + wild-type P66 expressed from pBSV2G (B313+*p66^cp^*) did not react with the anti-c-Myc antibody using either live cells or permeabilized cells (**Figure 4**). This validates the specificity of the anti-c-Myc antibody for use in assessing the localization of the c-Myc tags in P66 proteins produced by *Borrelia burgdorferi*. Both B313-K487 and B313-C-term stained for c-Myc under permeabilized cell staining conditions, but only B313-K487 stained for c-Myc under live cell staining conditions. These controls replicate the previous study by Kenedy *et al*. that demonstrated K487 was surface exposed on *Borrelia burgdorferi* while the C-terminus was not (Kenedy et al., 2014).

B313-E33, B313-T187, B313-E334, and B313-N580 all stained positive for both c-Myc and flagellin under permeabilized cell staining conditions (**Figure 4**). B313-T187, B313-E334, and B313-E33 stained positive for c-Myc when the cells were intact; however, the anti-c-Myc antibody only bound to a subset of cells for each strain. Since the B313 strain derivatives produce endogenous WT P66 along with the c-Myc-tagged mutants, the WT and mutant forms of P66 are in competition within the cells for production, oligomerization, and localization on the outer membrane. Therefore, it is possible that in some cells the endogenous WT P66 outcompetes the c-Myc-tagged mutant P66 forms for outer membrane localization. B313-N580 did not stain for c-Myc under live cell staining conditions. Repeated attempts to generate the B313-D303 strain failed, so we were unable to determine whether the conserved D303 domain is surface exposed although it is predicted to be periplasmic. N580 was shown by the Proteinase K assay not to localize to the outer membrane which is consistent with the lack of staining under live cell conditions. Taken together, immunofluorescence of the live cells demonstrated that E33, T187, E334, and K487 were extracellular, whereas the C-term was not surface exposed, all in accordance with the predicted model (**Figure 1**).

### c-Myc-tagged P66 mutants exhibit various oligomeric states

**Figure 5:**
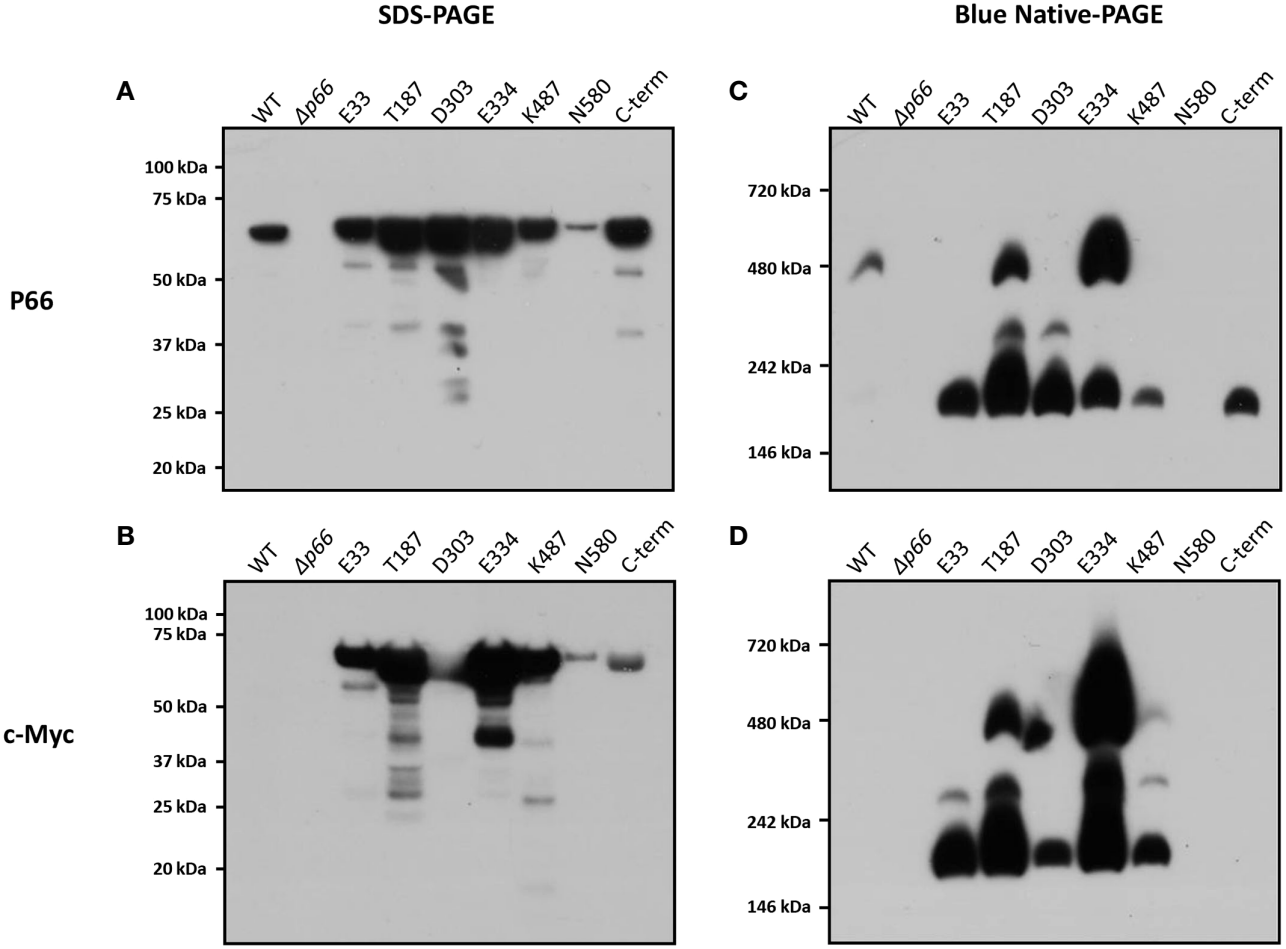
BN-PAGE, SDS-PAGE, and western blot analyses of P66 and the c-Myc tag reveal oligomerization state of B31 A3 c-Myc P66^cp^ strains. The outer membrane fractions of *B*. *burgdorferi* strains were subjected to SDS-PAGE **(A, B)** or to BN-PAGE **(C, D)**. Immunoblot analyses were performed using anti-P66 **(A, C)** or anti-c-Myc antibodies **(B, D)**.

Membrane proteins from *Borrelia burgdorferi* were isolated as described previously (Magnarelli et al., 1989). The membrane proteins (B-fractions) from *Borrelia burgdorferi* strains producing P66 with c-Myc insertions (B31 A3 c-Myc P66^cp^) were run on both SDS- and BN-PAGE and probed with anti-P66 and c-Myc antibodies (**Figure 5**). Under denaturing conditions, all of the c-Myc P66 proteins were detected at 66 kDa when the blot was probed using anti-P66 or anti-c-Myc antibody, and it is evident that expression of *p66* from the plasmid pBSV2G results in overproduction of the protein, as previously reported (Ristow et al., 2012). The level of B31 A3-N580^cp^ was considerably lower when compared to the levels of the other proteins. Under native conditions, WT P66 migrated to approximately 480 kDa, which corroborates previous studies suggesting formation of higher-order oligomers (Barcena-Uribarri et al., 2013). When the blot was probed using anti-P66 antibodies, a portion of B31 A3-T187^cp^ migrated to approximately 480 kDa while the remaining protein migrated to approximately 300 kDa and 200 kDa. A portion of B31 A3-E334^cp^ also migrated to 480 kDa and a portion migrated to approximately 200 kDa. None of the B31 A3-D303^cp^ protein migrated to 480 kDa, but portions did migrate to approximately 300 kDa and 200 kDa. B31 A3-E33^cp^, B31 A3-K487^cp^, and B31 A3-C-term^cp^ all migrated to approximately 200 kDa. However, when the BN-PAGE blot was probed with a monoclonal anti-c-Myc antibody, B31 A3-K487^cp^ was detected at 480 kDa, 300 kDa, and 200 kDa and B31 A3-E33^cp^ was detected at 300 kDa and 200 kDa. B31 A3-C-term^cp^ was not detected when the blot was probed with an anti-c-Myc antibody. B31 A3-N580^cp^ was not detected on either of the BN-PAGE blots. One possible explanation is that B31 A3-N580^cp^ appears relatively insoluble in non-ionic detergent (**Figure 2**), consistent with our other results indicating it is mis-localized. Refer to **Table 1** for a summary of our results.

**Table 1:** Data summary.

Strain	Conserved	Localization of Protein		Localization of Domain		Integrin-binding	Oligomer	Porin Function		Infectivity		
		Fraction	Prot. K Sensitive	PRED-TMBB	Immuno-fluorescence	ELISA	BN-PAGE	BLB(8-11 nS)	Vanco Suscept.	Short-term infection	28 day infection	ID_50_
WT	NA	Sup	Yes	NA	NA	NS	480 kDa	Yes	S	NS	5/5	2.4 x 10^1^ -6.8x 10^3^ (Ristow et al., 2012)
Δ*p66*	NA	NA	NA	NA	NA	NS	NA	No	R	NS	0/5	>10^8^ (Ristow et al., 2012)
P66^cp^ or P66^cc^	NA	Sup	Yes	NA	NA	ref	NT	Yes	S	ref	NT	1.8x10^4^ - 4.6x10^4^ (Ristow et al., 2012)
c-Myc E33	Conserved	Sup	Yes	EC	EC	NS	300, 200 kDa	No	I	NS	1/10	6.76x10^5^
c-Myc T187	Unique to RF	Sup	Yes	EC	EC	NS	480, 300, 200 kDa	Yes	S	> P66^cp^ in heart	6/10	NT
c-Myc D303	Conserved	Sup	Yes	PP	NT	NS	300, 200 kDa	No	I	NT	2/10	1.5x10^5^ - 3.2x10^5^
c-Myc E334	Unique to LD	Sup	Yes	EC	EC	NS	480, 300, 200 kDa	Yes	S	> P66^cp^ in heart	7/10	NT
c-Myc K487	EC control	Sup	Yes	EC	EC (+control) (Kenedy et al., 2014)	NS	480, 300, 200 kDa	Yes	S	NT	10/10	NT
c-Myc N580	Unique to LD	Pellet	No	EC	Not EC	NS	NA	Yes	Impaired growth	NT	NT	NT
c-Myc C-term	PP control	Sup	Yes	PP	Not EC (-control) (Kenedy et al., 2014)	> P66^cp^	200 kDa	Yes	S	NT	10/10	NT

EC, extracellular; PP, periplasmic; NA, not applicable; NT, not tested here; NS, not significant; S, sensitive; I, intermediate; R, resistant; ref, reference strain.

### c-Myc-tagged P66 mutants do not exhibit decreased ability to bind integrin

P66 is known to be an adhesin that binds β-chain integrins which facilitates dissemination during infection (Coburn et al., 1993; Coburn et al., 1994; Coburn et al., 1998; Coburn et al., 1999; Defoe and Coburn, 2001; Coburn and Cugini, 2003; Ristow et al., 2015). Therefore, we hypothesized that integrin-binding capabilities were not attenuated in the c-Myc mutants. Because B31 A3 produces numerous adhesins causing high background binding, we cloned the c-Myc constructs into the high passage, noninfectious HB19 strain. These constructs were cloned in the pBSV2G plasmid in the HB19 KO4 3-8B (Δ*p66*) background (Coburn and Cugini, 2003). Purified integrin was immobilized on a 96-well plate and incubated with spirochetes. The plates were washed to remove unbound bacteria and DNA was extracted from the wells. qPCR was performed to quantify the number of genomes bound to each well. Based on previous studies (Coburn and Cugini, 2003), we expected that the Δ*p66* strain would exhibit significantly reduced binding to integrin relative to the WT and the WT allele-complemented (Δ*p66*+vector+WT) controls. Previous studies measured recombinant P66 to bind α_v_β_3_ integrin with a K_D_ of 0.13 µM by surface plasmon resonance (Ristow et al., 2015) and the Δ*p66* strain to bind with ≤10% efficiency of the WT (Coburn and Cugini, 2003). However, this difference was not observed in the present work (**Supplementary Figure S1**). In contrast, the HB19-C-term strain exhibited statistically significant binding above the HB19-WT strain. No c-Myc P66^cp^ strain exhibited detectably decreased integrin-binding relative to controls.

### c-Myc-tagged E33 and D303 P66 mutants exhibit altered porin function

**Figure 6:**
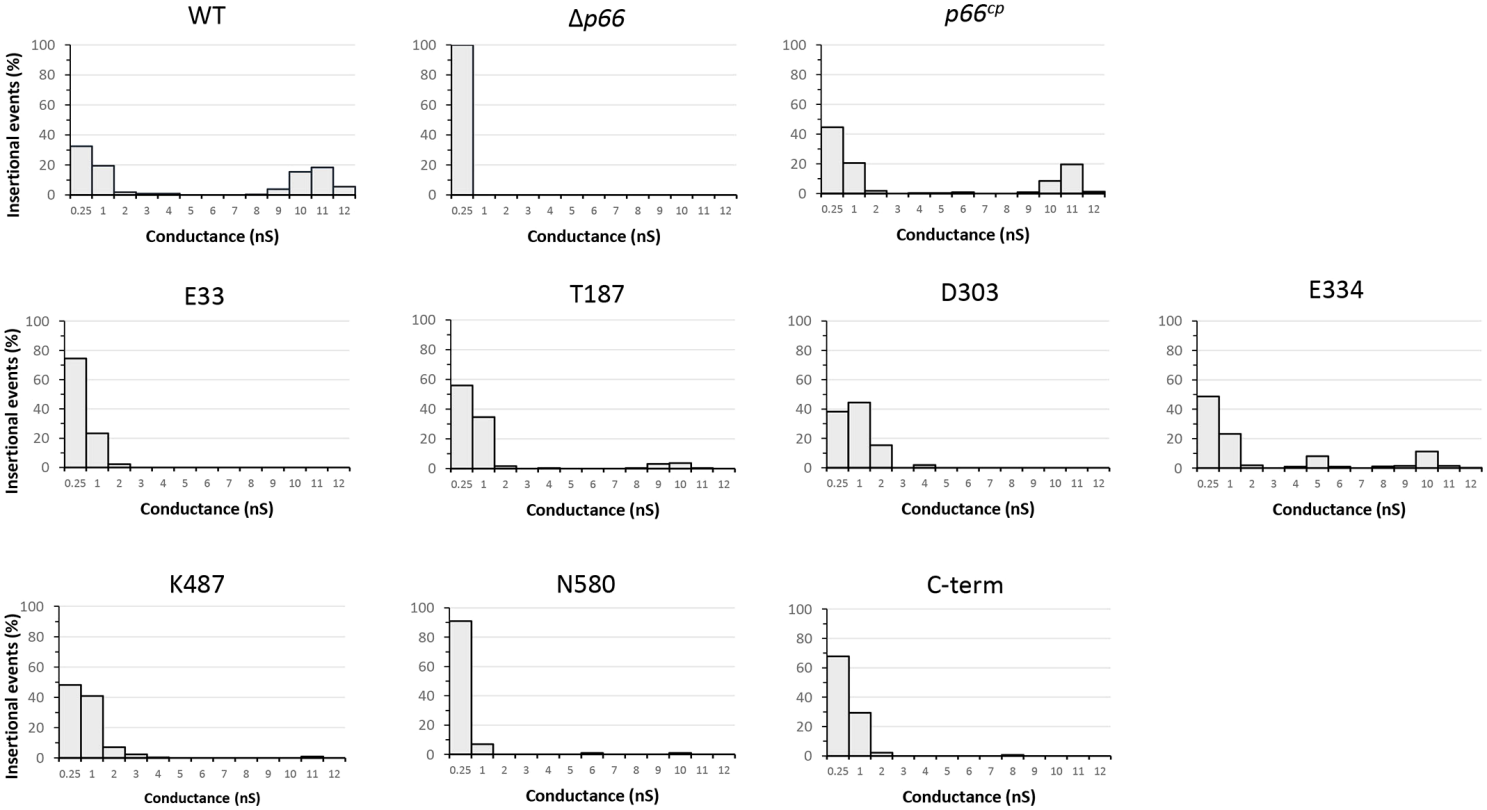
Black lipid bilayer analyses reveal different porin activities of outer membrane fractions of strain *B*. *burgdorferi* B31 A3 WT, Δ*p66*, and c-Myc P66^cp^ derivative strains. The histograms of the single-channel conductance distributions were obtained from at least 100 insertional events derived from at least three different membranes. Outer membrane fractions were diluted in 1% Genapol and added to the lipid bilayer in 1 M KCl. The applied voltage was 20 mV. The experiment was performed in duplicate (or in triplicate for C-term) for all strains with one representative shown. The conductance was measured in nano-Siemens (nS).

In black lipid bilayer assays, P66 exhibits channel-forming activity with a conductance of ~11 nS (Skare et al., 1997; Pinne et al., 2007; Barcena-Uribarri et al., 2013; Kenedy et al., 2014). The high conductance is the result of 7-8 monomeric subunits of P66 each with a ~1.5 nS conductance (Barcena-Uribarri et al., 2013). The black lipid bilayer assays performed here corroborated previous finding that WT P66 forms a pore with a conductance of ~11 nS (**Figure 6**). The outer membrane fraction isolated from Δ*p66* did not produce any pores with conductance exceeding 0.25 nS, while the complementation of P66 restored the channel conductance of ~11 nS. The insertional events with smaller conductance values are most likely due to lower oligomeric states of P66 found in the B-fractions (Thein et al., 2012; Barcena-Uribarri et al., 2014). The outer membrane fractions from B31 A3-E33^cp^ and B31 A3-D303^cp^ did not produce pores with conductances exceeding 4 nS, suggesting possible effects on porin structure, oligomerization, or both. The outer membrane fractions from B31 A3-T187^cp^, B31 A3-E334^cp^, B31 A3-K487^cp^, and B31 A3-C-term^cp^ all produced pores with a conductance of between 8-11 nS. Although there were fewer insertional events in the 8-11 nS range for B31 A3-K487^cp^ and B31 A3-C-term^cp^ than for the B31 A3-T187^cp^ and B31 A3-E334^cp^, their presence does suggest that these mutants retained at least some porin function. The membrane proteins isolated from B31 A3-N580^cp^ produced mostly pores with a conductance of 0.25 nS and very few with a conductance of 6 and 10 nS in the B-fraction.

**Figure 7:**
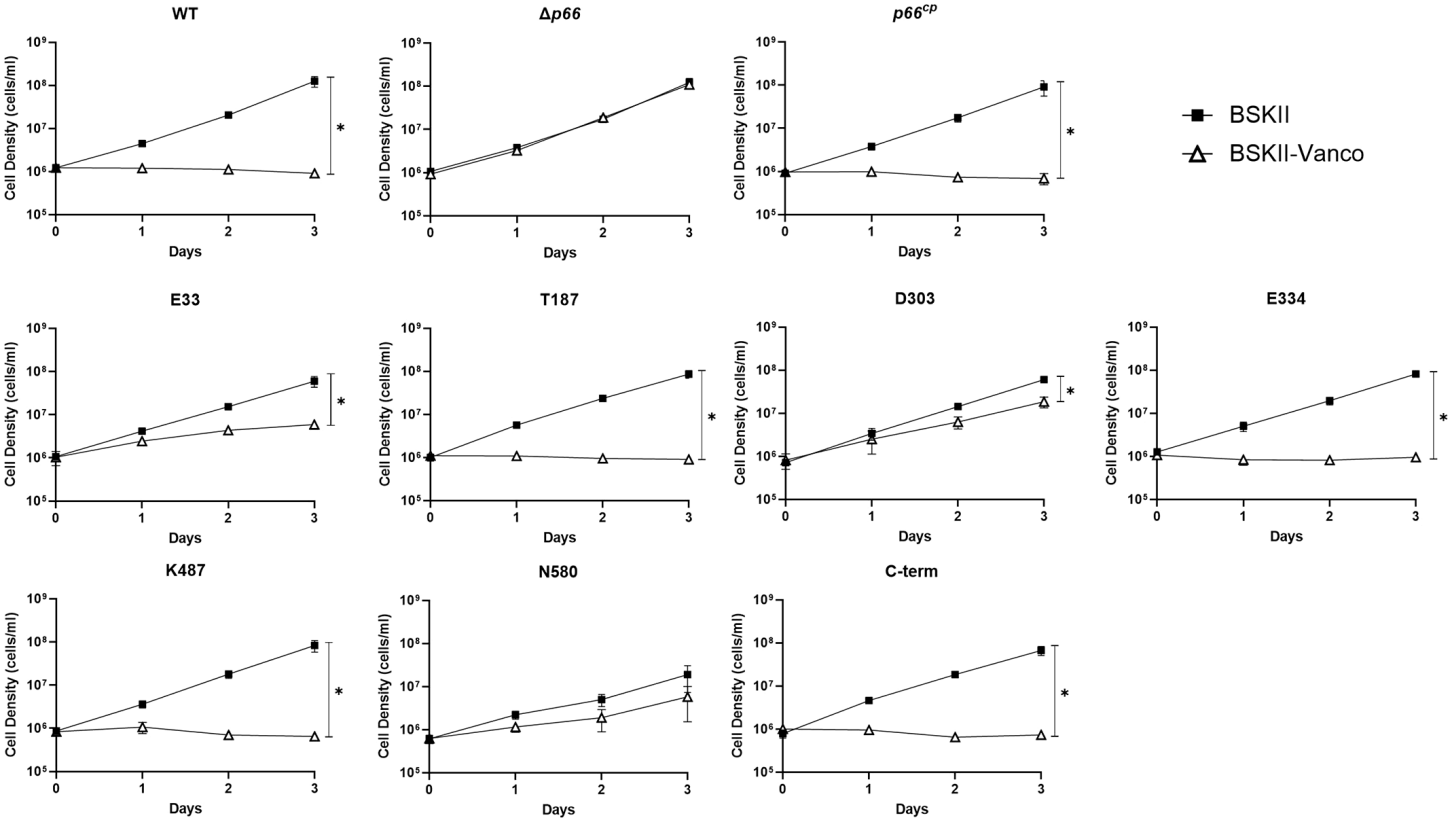
Vancomycin has differential effects on the growth of *B*. *burgdorferi* c-Myc P66^cp^ strains. B31 A3 WT, Δ*p66*, and c-Myc P66^cp^ strains were grown to exponential phase, diluted to 1 x 10^6^ cells ml^-1^ in BSKII and BSKII + vancomycin (1 μg ml^-1^) and incubated at 33°C. Cultures densities were determined daily. Data are presented as mean ± SEM of six replicates for WT, Δ*p66*, *p66^cp^* and three replicates for all the c-Myc P66^cp^ strains. Slopes were analyzed using linear regression (* = p< 0.05).

We used antibiotic susceptibility as a second method to assess P66 porin function. Vancomycin targets peptidoglycan synthesis, which occurs in the periplasm of Gram-negative bacteria, including *Borrelia burgdorferi* (Liu et al., 2009; Jutras et al., 2016; Wu et al., 2018). B31 A3 WT is susceptible to 1 µg ml^-1^ vancomycin, while Δ*p66* is resistant (**Figure 7**). Production of P66 from the pBSV2G plasmid in Δ*p66* (*p66^cp^*) restores the susceptible phenotype, demonstrating that P66 is necessary for vancomycin activity on *Borrelia burgdorferi* at this concentration. We assessed vancomycin susceptibility of the *Borrelia burgdorferi* strains producing c-Myc tagged P66. B31 A3-E33^cp^, B31 A3-T187^cp^, B31 A3-D303^cp^, B31 A3-E334^cp^, B31 A3-K487^cp^, and B31 A3-C-term^cp^ had a significant growth impairment when incubated in BSKII + vancomycin (1 µg ml^-1^) compared to untreated cultures. Although B31 A3-E33^cp^ and B31 A3-D303^cp^ had impaired growth in BSKII + vancomycin, they were able to replicate and displayed an intermediate vancomycin susceptibility phenotype (**Figure 7** and **Supplementary Table S6**). Both E33 and D303 mutants grew significantly better than WT, but significantly worse than Δ*p66* in vancomycin. These results were corroborated by the black lipid bilayer assay (**Figure 6**). The vancomycin assay was also performed on the B31 A3 c-Myc P66^cc^ strains with similar results (**Supplementary Figure S2** and **Supplementary Table S7**).

### c-Myc tagged E33 and D303 p66 mutants exhibit decreased infectivity in murine models

Due to the overproduction of P66 when produced from the pBSV2G vector and the loss of linear plasmid 25 (lp25) in all c-Myc P66^cp^ strains, the c-Myc P66^cp^ mutants were not appropriate for testing in traditional mouse infections (Ristow et al., 2012). Rather, to determine whether the c-Myc P66^cp^ mutants exhibited altered bacterial burdens in blood or tissues, we utilized a short-term infection model of hematogenous dissemination. In brief, mice were anesthetized and inoculated intravenously, and the bacteria were allowed to circulate for one hour. Subsequently, mice are perfused to dislodge nonadherent spirochetes and euthanized. Select tissues were harvested and prepared for DNA extraction and qPCR to quantify bacterial burdens. Only three mutants were tested in this model: E33, T187, and E334. E33 was chosen because it is conserved between both LD- and RF-causing species of *Borrelia*. T187 was chosen because it follows a section that is unique to RF species. E334 was chosen because it is unique to LD species. No significant differences were identified between any of the tested strains in the blood, lung, liver, bladder, spleen, ankle joint, or ear (**Supplementary Figure S3**). However, T187 and E334 were enriched in the heart relative to the control strain harboring the WT *p66* allele. However, the biological significance of this finding is unknown as the Δ*p66* control strain did not exhibit decreased adherence in any tissue relative to WT or other strains.

**Figure 8:**
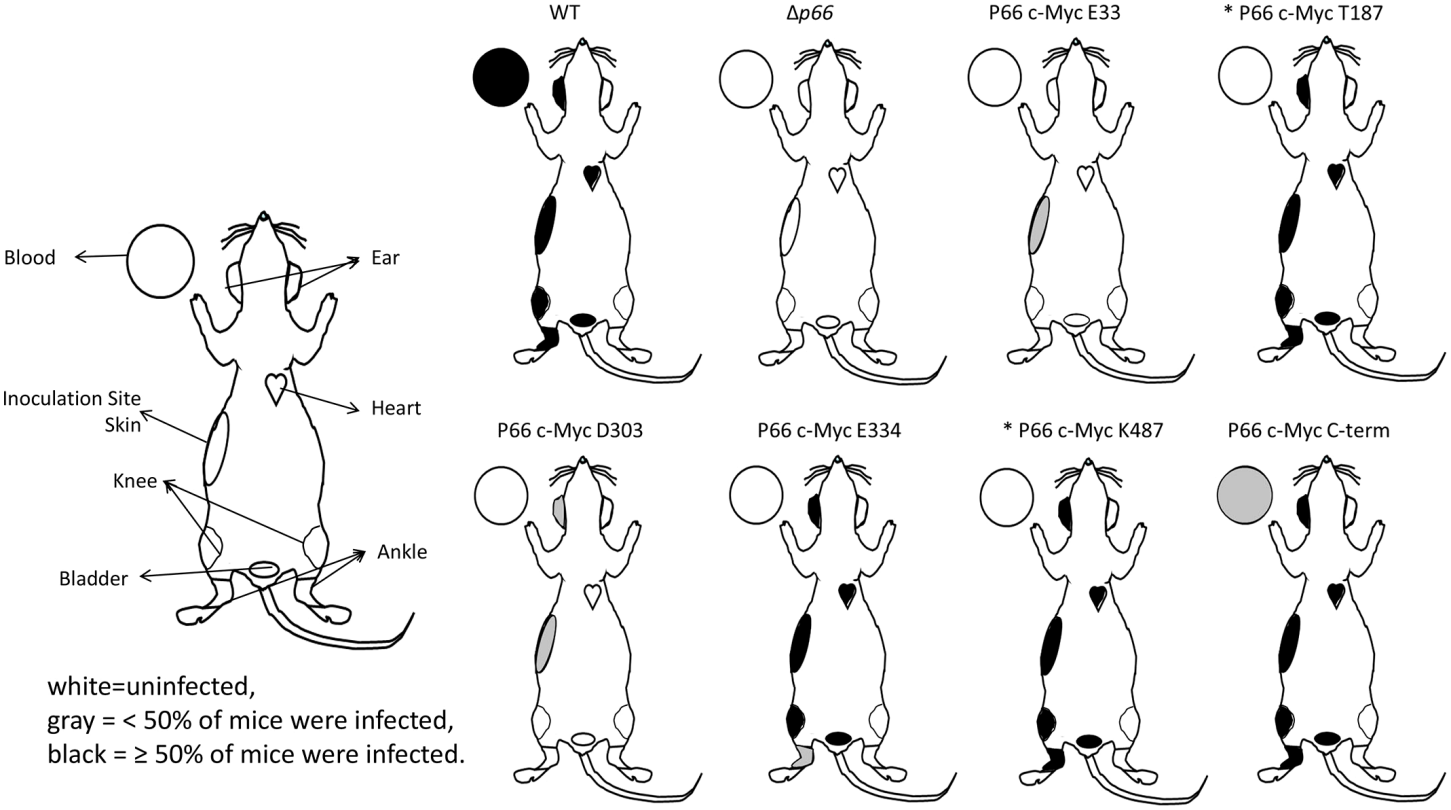
Infectivity and dissemination of B31 A3 c-Myc P66^cc^ mutants. Groups of 5 mice were inoculated with WT and mutant strains of *B. burgdorferi*. Two clones per c-Myc P66 mutant were included (10 mice per mutant). At 28 days post-infection, mice were euthanized and tissues were harvested. Tissues were used to inoculate BSKII medium and these cultures were monitored for spirochete growth for up to 8 weeks. Diagrams show the tissues included in the study and their culture positivity data. White = uninfected, gray = <50% of mice were infected, black = ≥50% of mice were infected. * = cultures were monitored for 1 week.

To determine whether the c-Myc mutations alter infectivity, we inoculated mice for a longer infection experiment. Since the existing c-Myc P66^cp^ strains were unsuitable for conventional infection experiments, the c-Myc mutants were re-cloned into an infectious B31 A3 background such that P66 was produced natively from the chromosome. N580 was excluded from the experiment because this mutant did not localize properly. These c-Myc P66^cc^ (complemented on the chromosome) strains were shown to produce c-Myc P66 (**Supplementary Figure S4**), localize P66 to the outer membrane (**Supplementary Figure S5 and Supplementary Table S8**), and exhibit porin function profiles (**Supplementary Figure S2** and **Supplementary Table S7**) comparable to the c-Myc P66^cp^ (complemented on the plasmid) strains except that E33 and D303 exhibited vancomycin-resistant rather than intermediate phenotypes. B31 A3 WT, Δ*p66*, vehicle (PBS with 0.2% normal mouse serum), and two clones per c-Myc mutant were inoculated subcutaneously with 1 x 10^5^ spirochetes per mouse in groups of five mice. Four weeks post-inoculation, mice were euthanized and tissues harvested and placed in BSKII medium. These cultures were monitored by darkfield microscopy for up to 8 weeks for the recovery of viable spirochetes. The cultures from infections with the T187 and K487 mutants were only able to be monitored for 1 week. However, a retrospective examination of the data for all the other strains indicated that no mice that were negative at the 1 week time point ever turned positive (**Supplementary Table S9**). Furthermore, only the WT (4 knees and 1 ear) and the c-Myc E334 mutants (2 bladders and 4 hearts) had any tissues that turned positive after the first week. Based on this, we are confident that the total number of infected mice is correct and only a few tissues were possibly missed for positivity. B31 A3 WT spirochetes were able to establish a disseminated infection in all of the tested mice and tissues (**Figure 8** and **Supplementary Table S9**). In contrast, Δ*p66* was completely noninfectious. Interestingly, B31 A3-T187^cc^, B31 A3-E334^cc^, B31 A3-K487^cc^, and B31 A3-C-term^cc^ also exhibited successful establishment of disseminated infection. In contrast, B31 A3-E33^cc^ and B31 A3-D303^cc^, the mutants harboring c-Myc insertions within conserved domains, were attenuated in their ability to establish infection or disseminate.

To quantify the attenuation of the B31 A3-E33^cc^ and B31 A3-D303^cc^ mutants in murine infection, we performed an ID_50_ experiment. The ID_50_ value for B31 A3-E33CC was 6.76x10^5^ spirochetes/mouse while the ID_50_ for B31 A3-D303CC was in the range of 1.5-3.2x10^5^ spirochetes/mouse (**Supplementary Table S10**). This is at least 1 log higher than previously published values for the WT and *p66^cc^* strains, which ranged between ~10^1^ and ~10^4^ (Ristow et al., 2012). While not completely noninfectious, these data demonstrate the importance of the E33 and D303 domains for P66 function(s) *in vivo*.

Some of the spirochetes that were re-isolated from infected mouse tissues were also subjected to western blots to test for c-Myc production (**Supplementary Figure S6**). These isolates all retained c-Myc tagged P66 signal, demonstrating that the recovered isolates retained c-Myc tagged P66 throughout infection.

## Discussion

The aim of this study was to identify P66 domains that are exposed on the surface of *Borrelia burgdorferi* and to begin to identify protein domains that have a role in protein localization, oligomerization, porin function, and infectivity. There is strong experimental evidence that suggests P66 has a β-barrel configuration in the bacterial outer membrane (Kenedy et al., 2014). However, since there is not a crystal structure of P66 and it is unique to the *Borrelia* genus, little more has been experimentally determined regarding the structure it forms in the bacterial outer membrane.

The alignment of sequences from LD-causing and RF-causing *Borrelia* species revealed some potentially interesting protein domains. First, there were several domains in which amino acids were highly conserved among all *Borrelia* species examined. Evolutionary amino acid conservation is a strong indicator of structural or functional importance (Karlin and Brocchieri, 1996; Capra and Singh, 2007; reviewed in: Chothia and Lesk, 1986). Beta-strands are less tolerant of mutations than helices, rendering beta-strands more likely to show high evolutionary conservation of amino acids in domains critical to protein function (Sitbon and Pietrokovski, 2007; Abrusan and Marsh, 2016). Therefore, we set out to determine where these conserved domains were located relative to the outer membrane, and whether they are important for P66 protein function and *Borrelia burgdorferi* infectivity. According to the PRED-TMBB prediction, some of the highly conserved domains are located on surface exposed loops and others span transmembrane domains and periplasmic loops. E33 is an example of a conserved amino acid in a domain predicted to be on a surface exposed loop, while D303 is an example of a conserved amino acid in a domain predicted to be periplasmic (**Figure 1**). There were several domains that contained ~5-10 amino acid insertions or deletions when LD *Borrelia* sequences were compared to RF *Borrelia* P66 sequences (e.g. T187, E334, K487, and N580). Amino acid insertions or deletions are more likely to occur on non-structured loops than in transmembrane regions and could possibly be involved in additional non-porin functions of P66 (Pascarella and Argos, 1992). We introduced c-Myc epitope tags into both conserved domains and insertion/deletion domains to begin to determine P66 topology and assess potential roles in P66 localization, oligomerization, and/or function.

The only c-Myc epitope tag insertion that interfered with P66 localization to the bacterial outer membrane was adjacent to N580. B31 A3-N580^cp^ was not cleaved by Proteinase K (**Figure 3**) indicating that the protein is not surface exposed on *Borrelia burgdorferi*. Additionally, the majority of c-Myc-N580 was not present in the soluble (supernatant) fraction of the octylglucoside extract (**Figure 2**) or in the B-fraction (membrane fraction) (**Figure 5**). This suggests that the amino acid domain encompassing N580 is vital to correct localization of P66 in the bacterial outer membrane. It is possible that this mutation interferes with the C-terminal β signal found in β-barrel proteins in other Gram-negative bacteria and mitochondria (reviewed in: Tommassen, 2010). The other c-Myc insertions did not affect P66 outer membrane localization.

The c-Myc epitopes inserted at E33, T187, and E334 are all surface exposed as determined by immunofluorescence microscopy (**Figure 4**) matching the PRED-TMBB predicted localization for each of these amino acids (**Figure 1**). The domain encompassing amino acids 181-187 (T187) has previously been described as important to integrin binding by P66 (Defoe and Coburn, 2001; Ristow et al., 2015), but this study is the first to experimentally demonstrate exposure on the bacterial surface. While P66 has been shown to bind to one host protein family (i.e. integrins), other *Borrelia burgdorferi* proteins are known to have multiple binding partners. BBK32, another adhesin produced by *Borrelia burgdorferi*, binds to fibronectin, glycosaminogylcans, and complement component C1 through three distinct domains (Probert et al., 2001; Lin et al., 2015; Garcia et al., 2016). Since high amino acid conservation may be an indicator of a domain important for protein structure or function and the highly conserved domain encompassing E33 is extracellular, it is an interesting candidate host interaction domain of P66. The E334 domain is highly conserved among LD-causing *Borrelia*, and therefore may be of interest in further investigations of the comparative pathogenic mechanisms of the two clades of *Borrelia*. One speculative possibility is that the E334 domain has a specific function for LD-causing *Borrelia.* Neither the 18 amino acids that comprise the conserved E33 domain (**Figure 1A**) nor the 16 amino acids that comprise E334 domain (**Figure 1A**) belong to any identified conserved domains in NCBI Conserved Domain^2^ database. Future experiments will be required to determine whether either the E33 or E334 domain has an *in vivo* function.

P66 oligomerizes into a heptamer or an octamer to form a complex detected at ~480 kDa with BN-PAGE that has a conductance of ~11 nS (Skare et al., 1997; Pinne et al., 2007; Barcena-Uribarri et al., 2013; Kenedy et al., 2014). The ~11 nS conductance is the additive combination of the seven or eight P66 subunits or monomers that form individual channels with ~1.5 nS conductance (Barcena-Uribarri et al., 2013). In this study, the BN-PAGE assay demonstrated that only protein complexes formed by WT, B31 A3-T187^cp^, B31 A3-E334^cp^, and B31 A3-K487^cp^ migrated to ~480 kDa (**Figure 5**), suggesting that these proteins form the previously reported P66 heptamers or octamers. Complexes formed by B31 A3-E33^cp^ and B31 A3-D303^cp^ did not migrate to ~480 kDa, but to ~300 kDa and ~200 kDa, which suggests that these proteins may form lower order oligomers than WT P66. This may indicate that the conserved domains around E33 and D303 are important for correct oligomerization of P66. B31 A3-C-term^cp^ migrated to ~200 kDa suggesting that the C-terminus of P66 is also important for proper oligomerization of P66. In addition, B31 A3-C-term^cp^ was detected in the BN-PAGE blot only with anti-P66 antibody suggesting that the c-Myc epitope tag may be structurally hidden or blocked from antibody detection in B31 A3-C-term^cp^ native conformation. The immunofluorescence staining of B313-C-term corroborated this result, as B31 A3-C-term^cp^ did not stain as brightly as B313-K487 or the other c-Myc insertions under permeabilized cell conditions (**Figure 4**). Aberrant oligomerization may also be due, at least in part, to general physiological disruption caused by overproduction of the proteins.

The black lipid bilayer assay was performed to assess the porin functions of the P66 proteins containing c-Myc insertions. Membrane proteins (B-fractions) from WT and *p66^cp^* each produced a channel with an ~11 nS conductance corresponding to the activity of P66 (**Figure 6**). The WT and Δ*p66* strains used for the black lipid bilayer assay contained lp25, while the strains harboring the pBSV2G vector did not (**Supplementary Table S2**). However, lp25 is not known to encode any proteins that might affect the black lipid bilayer results observed for *Borrelia burgdorferi* (Pinne et al., 2007; Ristow et al., 2015). B-fractions from B31 A3-T187^cp^, B31 A3-E334^cp^, and B31 A3-K487^cp^ each showed ~11 nS conductance (**Figure 6**) although there were fewer insertional events at ~11 nS for B31 A3-K487^cp^. In contrast, the B-fractions of B31 A3-E33^cp^, B31 A3-D303^cp^, and B31 A3-C-term^cp^ did not show 11 nS conductance, consistent with the BN-PAGE results that demonstrated those three P66 proteins did not oligomerize into a heptamer or an octamer either (**Figure 5**). The B-fractions of all of the *Borrelia burgdorferi* strains except Δ*p66* formed channels with a conductance ~1-2 nS (**Figure 6**). This smaller conductance is likely due to porins other than P66 or low-order P66 oligomers.

We developed a novel, second method to determine whether any of the c-Myc epitope tags disrupted P66 porin function. Porins are a major route of entry into the periplasm of Gram-negative bacteria for hydrophilic antibiotics (reviewed in: Fernandez and Hancock, 2012). Therefore, we sought to develop an assay that utilizes cell wall-active antibiotic susceptibility as a readout for screening P66 porin function. Vancomycin was an attractive candidate antibiotic because it is active in the periplasm and kills bacteria by binding to the Lipid II precursor at the C-terminal D-Ala-D-Ala building blocks of peptidoglycan (Liu et al., 2009). Typically, vancomycin is not highly effective against diderm bacteria because it cannot penetrate the outer membrane to access sites of peptidoglycan synthesis, but it has been shown to effectively kill *Borrelia burgdorferi in vitro* with a minimal inhibitory concentration of 0.25 μg ml^-1^ (Sharma et al., 2015).

While Δ*p66* is resistant to 1 µg ml^-1^ vancomycin, both WT and *p66 ^cp^* are susceptible, demonstrating that P66 has a critical role in vancomycin efficacy (**Figure 7**). In contrast, ampicillin, a different peptidoglycan-targeting antibiotic that has a lower molecular weight than vancomycin, does not require the presence of P66 to kill *Borrelia burgdorferi* (J. Kessler and J. Coburn, unpublished). We screened our c-Myc P66 mutant producing strains to determine whether any were resistant to vancomycin. B31 A3-N580^cp^ grew significantly slower in BSKII regardless of the presence or absence of antibiotic compared to WT, suggesting a gross malfunction of normal *Borrelia burgdorferi* physiology (**Figure 7** and **Supplementary Table S6**). That was consistent with B31 A3-N580^cp^ not localizing to the surface of *Borrelia burgdorferi*, but was not necessarily expected since the Δ*p66* grows well *in vitro*. The strains producing T187, E334, and K487 c-Myc-tagged P66 mutants were susceptible to 1 µg ml^-1^ vancomycin, consistent with surface localization and at least some restoration of porin function as assessed in the black lipid bilayer assay. B31 A3-E33^cp^ and B31 A3-D303^cp^ both had intermediate phenotypes (**Figure 7** and **Supplementary Table S6**). They both grew significantly better than WT in the presence of 1 µg ml^-1^ vancomycin, but also significantly worse than Δ*p66*. In slight contrast, the B31 A3-E33^cc^ and B31 A3-D303^cc^ strains were both resistant to vancomycin. Moreover, the mutant phenotypes could not be accounted for simply by different *in vitro* growth rates in BSKII medium (**Supplementary Tables S6**, **S7**). Perhaps the overproduction of P66 in the c-Myc P66^cp^ strains causes an increased sensitivity to vancomycin. Nevertheless, the E33 and D303 mutants exhibit altered P66 porin function. The B31 A3-E33^cp^ and B31 A3-D303^cp^ results corroborated the black lipid bilayer assay results, again demonstrating that the c-Myc tag inserted into P66 in the highly conserved domains of E33 and D303 disrupted P66 porin formation and function. It is interesting that B31 A3-C-term^cp^ is similar to B31 A3-E33^cp^ and B31 A3-D303^cp^ in that it does not form a heptamer or an octamer and it does not produce channel with ~11 nS conductance, but B31 A3-C-term^cp^ is sensitive to vancomycin while B31 A3-E33^cp^ and B31 A3-D303^cp^ are more resistant (**Figure 7**). These data suggest that the oligomeric state of P66 is not critical to vancomycin entry into the cell, and that the c-Myc tag insertions at E33 and D303 may affect the monomeric channel structure of the porin.

The down-regulation of certain porins or the selection of mutations that affect a specific porin can lead to antibiotic resistance in Gram-negative bacteria (reviewed in: Fernandez and Hancock, 2012). *E. coli* mutants resistant to carbenicillin have reduced production of porin OmpF (Harder et al., 1981), and down-regulation of *oprD* in *Pseudomonas aeruginosa* results in carbapenem resistance (Wolter et al., 2004). A G to D mutation in loop L3 of OmpC/OmpF like protein from *Enterobacter aerogenes* resulted in multidrug resistance (De et al., 2001). While we do not fully understand the mechanisms underlying the role of P66 in *Borrelia burgdorferi* susceptibility to vancomycin, we have shown that mutations of P66 affecting porin function render the bacterium more resistant to *in vitro* killing by vancomycin.

We assessed the infectivity of *Borrelia burgdorferi* producing c-Myc-tagged P66 using two infection models. In a short-term model of hematogenous dissemination we observed some significant differences in the heart, but no other tissues tested. We also determined the effects of the c-Myc mutations in P66 on the infectivity of *Borrelia burgdorferi* in a traditional mouse model. This required restoration of the mutant *p66* alleles to the chromosomal locus (c-Myc P66^cc^). The N580 mutant was excluded because it did not localize properly to the outer membrane. The c-Myc P66^cc^ mutants were assessed for P66 protein production, outer membrane localization, and porin function prior to mouse infections. The porin function profiles of the c-Myc P66^cc^ strains were similar to those of their c-Myc P66^cp^ counterparts when assessed for P66 production and localization and vancomycin resistance.

The E33 and D303 mutants had the most severe infectivity defects (1/10 and 2/10 mice infected respectively) and poor dissemination (**Supplementary Table S9**). This coincided with elevated ID_50_ values (**Supplementary Table S10**). In contrast, T187 and E334 exhibited dissemination and higher infectivity (6/10 and 7/10 mice infected respectively). The K487 and C-term mutants exhibited infectivity and dissemination at WT levels (10/10 mice infected). Notably, the E33 and D303 c-Myc insertions interrupt domains that are well conserved between LD- and RF-causing species of *Borrelia* (**Figure 1**).

Known characteristics of P66 that might contribute to infectious phenotype observed in mice include the following: P66 production levels, oligomerization, integrin-binding, and porin function. The B31 A3-E33^cc^ and B31 A3-D303^cc^ strains were not deficient in P66 production relative to the other c-Myc P66^cc^ mutants (**Supplementary Figure S4**). We performed densitometry on stain-free gel images, western blots probed with a polyclonal α-P66 antibody, and western blots probed with a polyclonal α-c-Myc antibody. Densitometric comparisons of P66 produced by the mutants differed by method. While the stain-free gel showed comparable results for all the mutants, this method is less specific than immunoblotting. The α-P66 blot and α-c-Myc blots showed different patterns of P66 production. For instance, for the α-P66 blot, T187 had the highest production while E334 had the lowest. In contrast, for the α-c-Myc blot, E33 and E334 had the highest while D303 and C-term had the lowest. Other c-Myc antibodies have been shown to exhibit variable reactivity depending on the flanking protein sequences and it is suspected to be the case here (Schuchner et al., 2020). In any case, the E33 and D303 mutants did not produce P66 at levels less than the more infectious c-Myc-tagged P66 strains.

In terms of oligomerization, BN-PAGE results indicated that c-Myc E33^cp^ and c-Myc D303^cp^ mutant strains ran at ~200 kDa (potentially indicative of trimers) instead of the ~480 kDa signal seen for WT P66. c-Myc D303^cp^ also has a fainter signal at ~330 kDa (potentially indicative of pentamers) (**Figure 5**). It is unlikely that the oligomerization state of E33 and D303 accounts for the deficiency in infectivity because c-Myc K487^cp^ and c-Myc C-term^cp^ also lack the ~480 kDa signal yet remain fully infectious.

Because integrin-binding is the other known function of P66, we wanted to determine whether this function remains intact in the c-Myc mutants. Unfortunately, the success of this assay was hampered by the unavailability of previously described reagents, specifically Linbro 96-well plates (ICN) and integrins purified on the basis of activity (Coburn et al., 1998; Coburn et al., 1999). A further limitation to this experiment is the strain background that can be used. P66 integrin-binding assays are performed in a high passage *Borrelia burgdorferi* strain from the HB19 background because it encodes fewer adhesin proteins and increases the signal to noise ratio in the assay (Coburn and Cugini, 2003; Ristow et al., 2015). Thus, this experiment was performed on HB19 c-Myc P66^cp^ strains rather than the B31 A3 c-Myc P66^cc^ strains that were used for infections. Significant differences were not detected between the WT and Δ*p66* control strains. Only the C-term mutant bound significantly more than the Δ*p66* + vector + WT (*p66^cp^*) control strain (**Supplementary Figure S1**). Therefore, this assay does not have a dynamic range that allows us to detect differences in integrin-binding between the majority of the strains. Other approaches to measure P66 integrin-binding may yield clearer results. Previous work has measured integrin-binding by surface plasmon resonance (i.e. recombinant P66 with purified integrins) or use of radiolabeled bacteria and purified integrins, recombinant P66 with integrin-producing mammalian cells, or *p66* mutant strains with integrin-producing mammalian cells (Coburn et al., 1998; Coburn et al., 1999; Coburn and Cugini, 2003; Ristow et al., 2015). However, it is notable that none of the c-Myc mutants exhibited significant losses in integrin-binding activity. Even if integrin-binding were diminished but not detected in these strains, integrin-binding and porin function may very well be independent as integrin-binding deficient *p66* mutants retain typical P66 channel conductance and integrin-binding itself is not essential for infectivity (Ristow et al., 2015).

Taken together, this work sheds new light on P66, which is a critical protein for *Borrelia* spirochetes to cause infection in mammals. While it is possible that the presence of the c-Myc sequence, independent of insertion location, may be altering P66 activities, our data suggest that it is the location of where the c-Myc sequence is inserted that alters P66 activity due to our internal controls. For instance, while c-Myc-E33 and c-Myc-D303 do not produce conductance higher than 4nS, the other c-Myc-tagged P66 forms do produce conductance between 8-11nS similar to WT P66 (**Figure 6**). Additionally, the B31 A3-E33^cc^ and B31 A3-D303^cc^ mutants displayed attenuated infectious phenotypes in mice while B31 A3-K487^cc^ and B31 A3-C-term^cc^ mutants displayed infectious phenotypes similar to WT (**Figure 8** and **Supplementary Table S9**). We developed a novel vancomycin-based screen for P66 porin function that was validated by the black lipid bilayer experiments. We further identified P66 domains that are conserved or unique between LD- and RF-causing species and implicated them in surface localization, oligomerization, porin function, and infectivity. The porin function defects in E33 and D303 were unique in the panel of c-Myc mutants (**Figures 6**, **7**, and **Supplementary Figure S2**). This evidence suggests that the porin function of P66 is important *in vivo*. However, these mutants do not recapitulate the complete loss of infectivity of the Δ*p66* strain. Therefore, P66 porin function likely contributes significantly to infectivity, but it alone is not sufficient to fully account for the infectivity phenotype of the Δ*p66* mutant, as was the case for the integrin-binding deficient mutants (Ristow et al., 2015). Furthermore, this work suggests the importance of conserved domains and retention of P66 porin function during infection.

## Data availability statement

All data presented in this study can be found in the article/**Supplementary Material**.

## Ethics statement

The animal study was reviewed and approved by the Medical College of Wisconsin Institutional Animal Care and Use Committee.

## Author contributions

MC contributed conceptualization of the project, generation and characterization of strains, execution of experiments, data analysis and interpretation, and drafting the manuscript. CF generated and characterized strains, executed experiments, analyzed and interpreted data, and assisted in drafting the manuscript. BH performed mouse experiments and data analysis, JK generated strains and executed experiments, and PA performed mouse experiments and data analysis. MS, MV-C, MB, and JC also performed experiments and data analysis. MC, CF, MS, JC, SB, BH, JK, and PA contributed to editing. JZ provided purified integrin. JC and SB contributed conceptualization of the project and funding acquisition. All authors contributed to the article and approved the submitted version.

## Funding

This work was funded by grants R01 AI084873, R01 AI118799, R01 AI121401, R21 AI140510, and R21 AI147573 from the National Institutes of Health, National Institute of Allergy and Infectious Diseases to JC and a grant from the Swedish Research Council to SB.

## Acknowledgments

We thank Drs. Darrin Akins and Melisha Kenedy of the University of Oklahoma for generously providing anti-BB0405 and anti-OppAIV, and for publishing their previous work on using the c-Myc tag to explore P66 structure in the outer membrane of *Borrelia burgdorferi*. We also thank Dr. Hiromi Sato for sharing expertise in development of the immunofluorescence microscopy protocol, and Dr. Yngve Östberg and Ingela Nilsson for technical assistance.

## Conflict of interest

The authors declare that the research was conducted in the absence of any commercial or financial relationships that could be construed as a potential conflict of interest.

## Publisher’s note

All claims expressed in this article are solely those of the authors and do not necessarily represent those of their affiliated organizations, or those of the publisher, the editors and the reviewers. Any product that may be evaluated in this article, or claim that may be made by its manufacturer, is not guaranteed or endorsed by the publisher.

## Footnotes

1
http://bioinformatics.biol.uoa.gr/PRED-TMBB

2
https://www.ncbi.nlm.nih.gov/Structure/cdd/wrpsb.cgi
