# Supplementary material for: Identification of amino acid domains of Borrelia burgdorferi P66 that are surface exposed and important for localization, oligomerization, and porin function of the protein
Source: Front Cell Infect Microbiol. 2022 Sep 23;12:991689. doi: 10.3389/fcimb.2022.991689 (PMC9539438; doi:10.3389/fcimb.2022.991689)
Supplement: Supplementary file 1 [file DataSheet_1.pdf]

Supplementary Material

1.1 Supplementary Figures

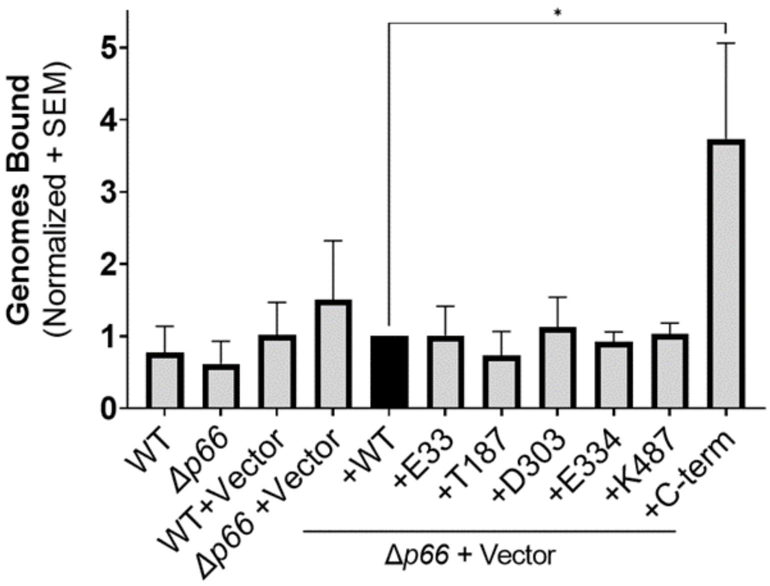

Supplementary Figure S1. Integrin-binding of HB19 c-Myc P66^{cp} mutants. Strains were grown to exponential phase, washed, and resuspended in HBSC buffer. The cells were inoculated into wells ± purified integrin $\alpha_v\beta_3$. The plates were centrifuged, incubated, and washed. DNA was purified and quantified by qPCR. Binding was normalized to the 'Δ*p66* + vector + WT *p66'* strain (black bar) as it is the isogenic control for the c-Myc P66^{cp} mutants. Data from technical replicates were combined for each independent experiment and the aggregate data from 3 independent experiments are shown. Statistical significance was detected by one-way ANOVA with Dunnett correction for multiple comparisons. * = $p<.05$.

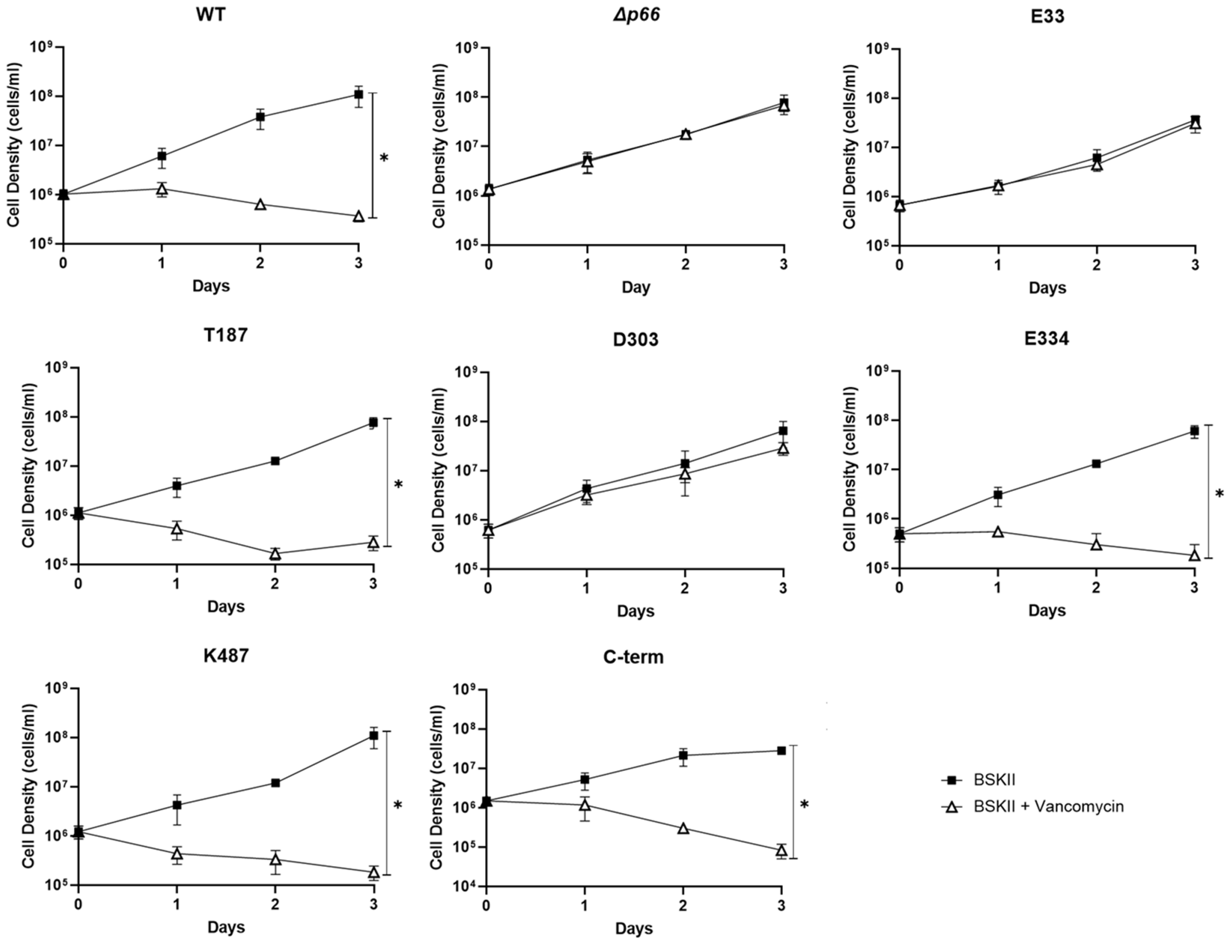

Supplementary Figure S2. Porin function of B31 A3 c-Myc P66^{cc} mutants. Exponential phase WT, Δ*p66*, and c-Myc *p66^{cc}* strains were cultured ± 1 µg ml^{-1} vancomycin. Culture densities for total cells were determined daily using a Petroff-Hausser counting chamber under darkfield microscopy. Data are presented as mean ± SEM of three independent replicates. Data were analyzed using simple linear regression in GraphPad Prism 9.2.0 (* = $p < 0.05$).

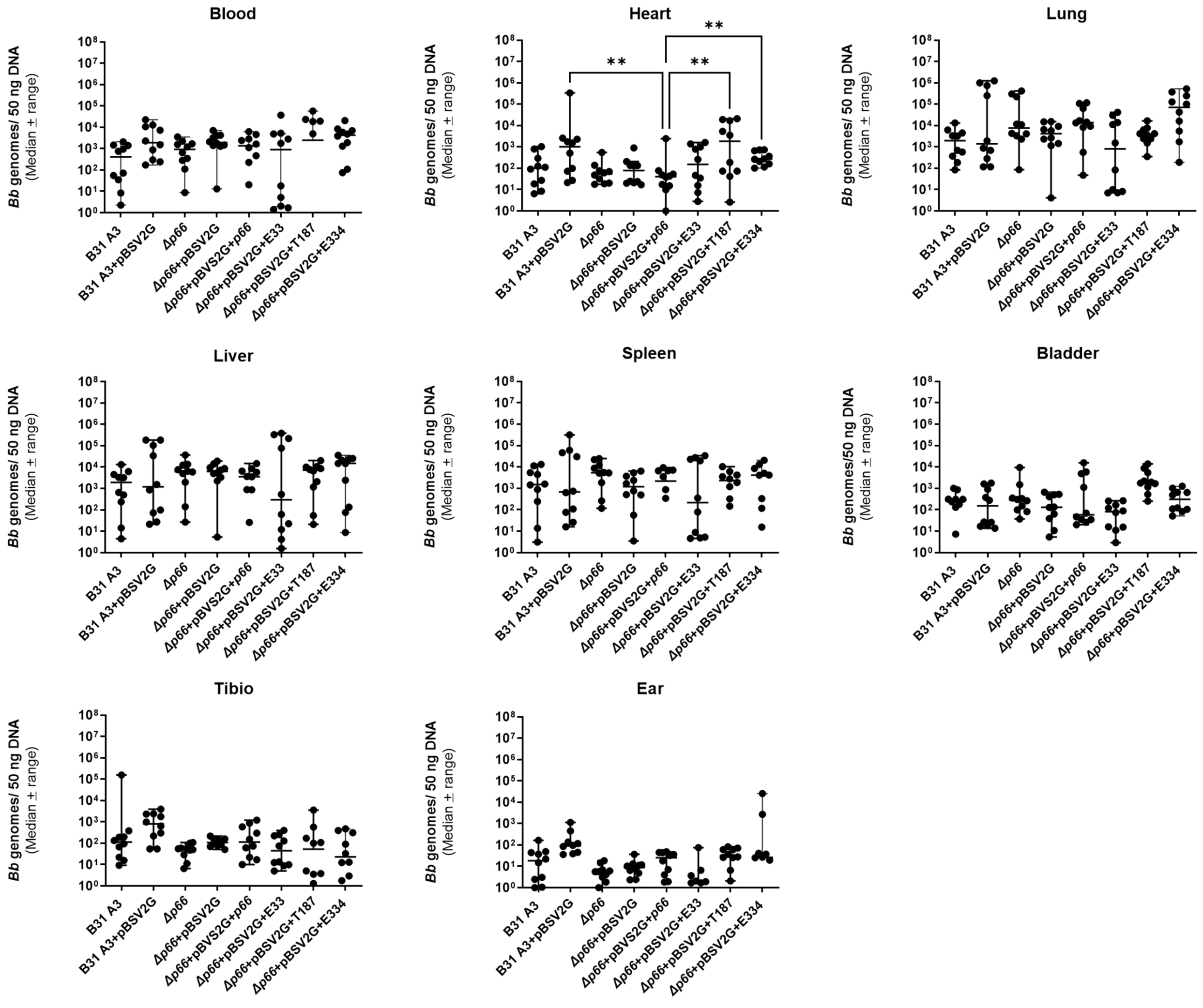

Supplementary Figure S3. Short-term infection model of B31 A3 c-Myc P66^{cp} strains. Mice were anesthetized and inoculated with $1x10^9$ cells ml^{-1} in 100 µl through the tail vein. After 1 hr, blood was obtained by cardiac puncture and the mice were perfused with saline. Following perfusion, lung, heart, liver, spleen, ear, and bladder tissues were collected and rinsed with PBS. An ankle joint (i.e. tibio-tarsal joint) was also collected. DNA was extracted from the tissues and bacterial burdens were quantified by qPCR. Each data point represents one mouse. Values were compared to the *Δp66*+pBSV2G+*p66* strain in a one-way ANOVA followed by a Kruskal-Wallis test. ** denotes $p<0.01$.

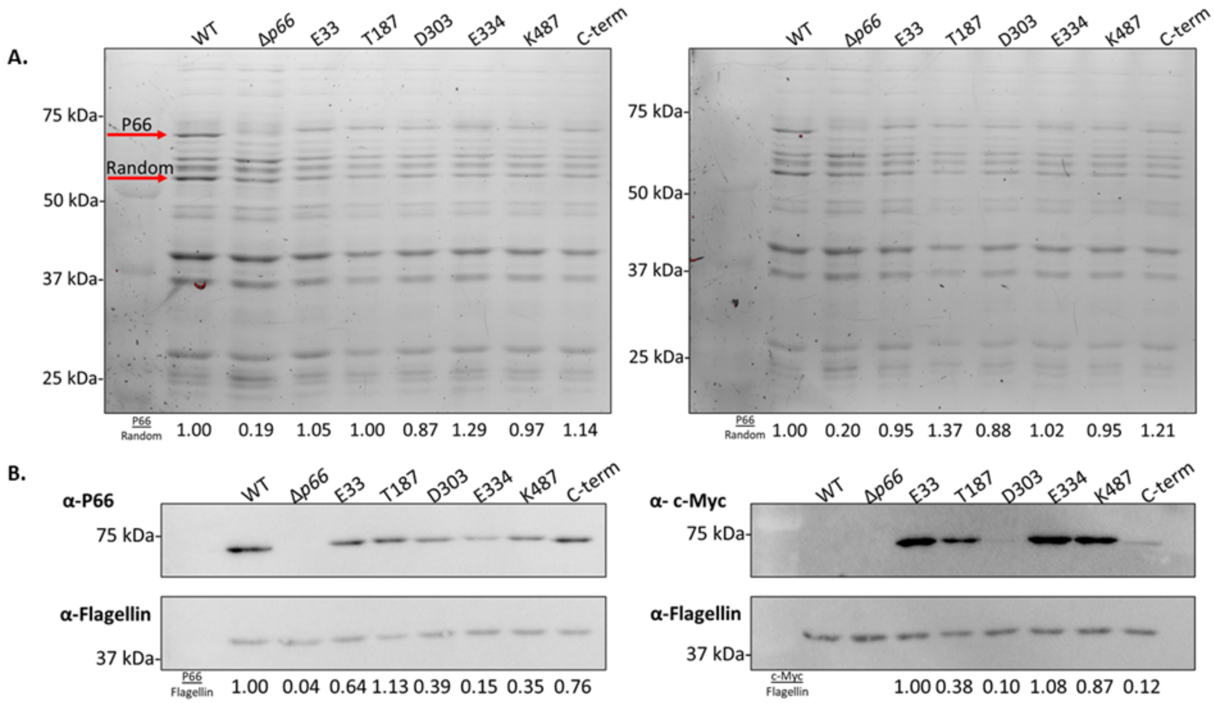

Supplementary Figure S4. P66 production in the c-Myc P66^{cc} strains. Cell lysates from B31 A3 WT, Δ*p66*, and c-Myc *p66^{cc}* strains were separated by SDS-PAGE. The inclusion of 2,2,2-Trichlorethanol in the polyacrylamide gels enabled the stain free imaging A) of 2 identically loaded gels. The P66 signal in each lane was determined relative to the WT P66 signal. Similarly a random band was selected as a loading control and signal for each lane compared to the WT. Relative densitometry was determined by dividing the relative P66 signal by the relative random band signal and is shown below the gel images. B) The gels were transferred to PVDF membranes and probed either with α-P66 and α-Flagellin or α-c-Myc and α-Flagellin. Anti-P66 and flagellin signals were each determined relative to the WT. The P66/Flagellin ratio was determined by dividing the relative P66 signal in a lane by its corresponding relative flagellin signal. Ratios are shown below the blots. Because the WT strain does not produce c-Myc P66, the α-c-Myc blot was instead normalized to the c-Myc E33 lane.

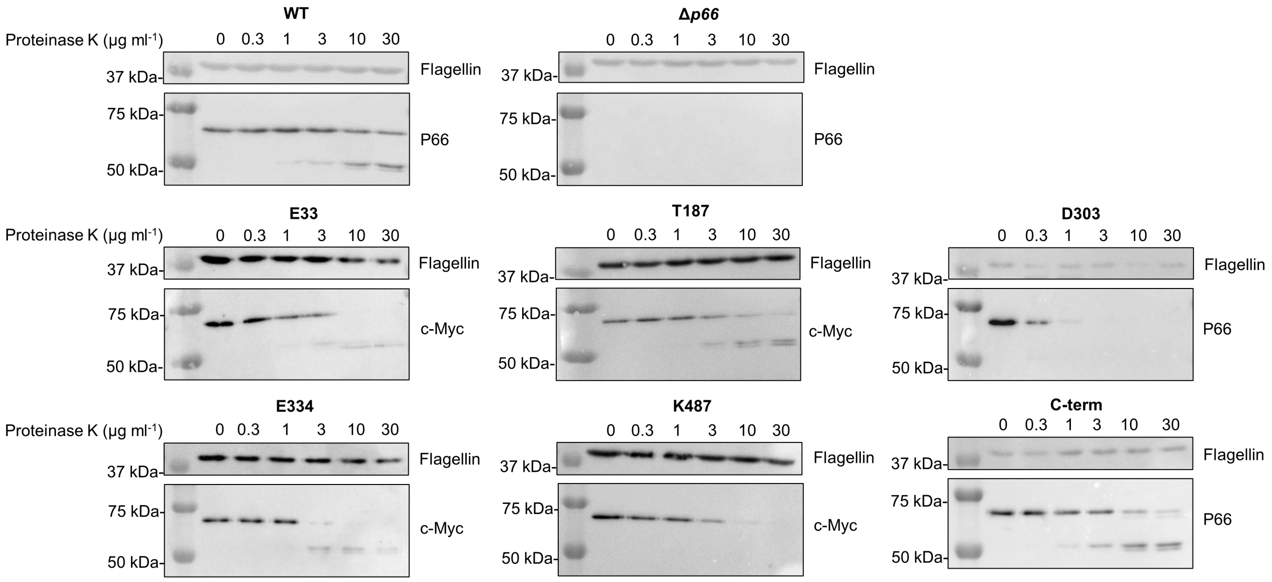

Supplementary Figure S5. Outer membrane localization of c-Myc P66[cc] mutants. Cells were incubated with increasing concentrations of Proteinase K. Following enzymatic digestion and deactivation of the Proteinase K, cell lysates were separated by SDS-PAGE, transferred to a PVDF membrane, and probed with antibodies to flagellin and P66 or c-Myc. Bands were visualized though chemiluminescence using a Bio-Rad Chemidoc.

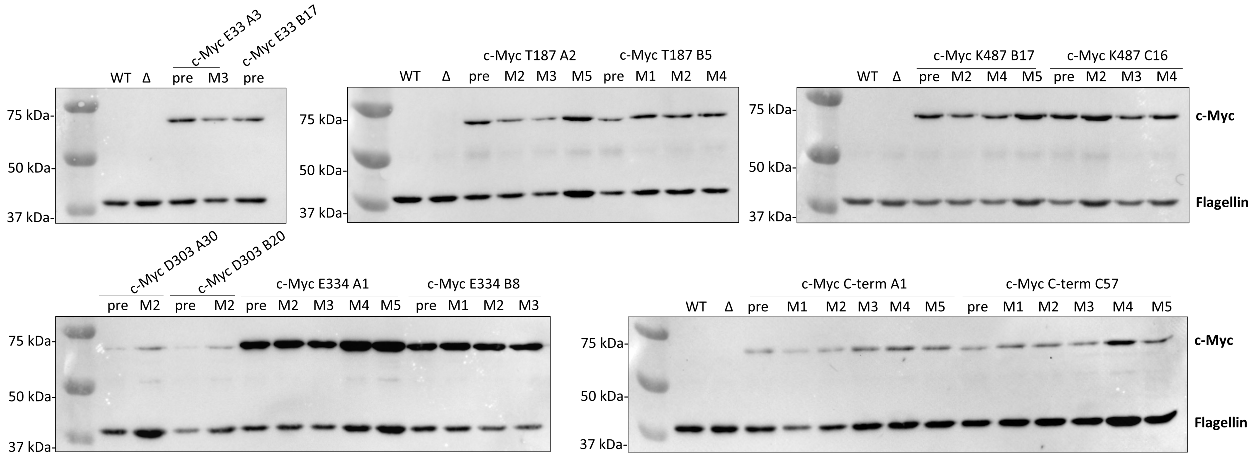

Supplementary Figure S6. B31 A3 c-Myc P66^{cc} mutants recovered post-infection retain the parental epitope tag. Lysates of *B. burgdorferi* recovered from different mice were collected and subjected to western blots. Shown are westerns blots of these samples that were probed with anti-c-Myc and anti-flagellin antibodies. Abbreviations are as follows: WT = wild type, Δ = Δ*p66*, pre = pre-infection parental strain, M1-M5 = individual mouse designations.

1.2 Supplementary Tables

Supplementary Table S1. *Escherichia coli* strains used in this study.

Strain Name	Description	Abx Markers	Reference
Top10	Used for cloning		Invitrogen Life Technologies (Carlsbad, CA)
DH5α	Used for cloning		
pGEMT Easy	Used for cloning	AmpR	Promega (Madison, WI)
pGTE p66 #7 Clone 1-1.4	Used as a PCR template for amplification of the GentR cassette	GentR	(Ristow et al., 2012)
Top10 Gibson Assembly (GA) myc E33 3-2	Harbors the pBSV2G + P66 c-Myc E33 plasmid	GentR	This Study
Top10 GA myc T187 3-1	Harbors the pBSV2G + P66 c-Myc T187 plasmid	GentR	This Study
Top10 GA myc D303 5-11	Harbors the pBSV2G + P66 c-Myc D303 plasmid	GentR	This Study
Top10 GA myc E335 2-5	Harbors the pBSV2G + P66 c-Myc E334 plasmid	GentR	This Study
Top10 GA myc K487 1-2	Harbors the pBSV2G + P66 c-Myc K487 plasmid	GentR	This Study
Top10 GA myc N580 3-3.2-1	Harbors the pBSV2G + P66 c-Myc N580 plasmid	GentR	This Study
Top10 GA myc C-term 2-3	Harbors the pBSV2G + P66 c-Myc C-term plasmid	GentR	This Study
DH5α + pBSV2G #2	Harbors the pBSV2G plasmid	GentR	This Study
XL-Gold P66 c-Myc E33 in pGEMT clone N	Harbors cloning Intermediate 1 for c-Myc E33: ligation of SacI and SphI double digested ‘pGEMT Easy’ and ‘Top10 Gibson Assembly (GA) myc E33 3-2’	AmpR	This Study
XL-Gold P66 c-Myc T187 in pGEMT clone AQ	Harbors cloning Intermediate 1 for c-Myc T187: ligation of SacI and SphI double digested ‘pGEMT Easy’ and ‘Top10 GA myc T187 3-1’	AmpR	This Study
XL-Gold P66 c-Myc D303 in pGEMT clone F	Harbors cloning Intermediate 1 for c-Myc D303: ligation of SacI and SphI double digested ‘pGEMT Easy’ and ‘Top10 GA myc D303 5-11’	AmpR	This Study
XL-Gold P66 c-Myc E334 in pGEMT clone A	Harbors cloning Intermediate 1 for c-Myc E334: ligation of SacI and SphI double digested ‘pGEMT Easy’ and ‘Top10 GA myc E335 2-5’	AmpR	This Study
XL-Gold P66 c-Myc K487 in pGEMT clone J	Harbors cloning Intermediate 1 for c-Myc K487: ligation of SacI and SphI double digested ‘pGEMT Easy’ and ‘Top10 GA myc K487 1-2	AmpR	This Study
XL-Gold P66 c-Myc C-term in pGEMT clone O	Harbors cloning Intermediate 1 for c-Myc C-term: ligation of SacI and SphI double digested ‘pGEMT Easy’ and ‘Top10 GA myc C-term 2-3’	AmpR	This Study
XL-Gold P66 c-Myc E33 in pGEMT clone F	Harbors cloning Intermediate 2 for c-Myc E33: insertion of GentR PCR product at MfeI site in Intermediate 1	AmpR, GentR	This Study
XL-Gold P66 c-Myc T187 in pGEMT clone B	Harbors cloning Intermediate 2 for c-Myc T187: insertion of GentR PCR product at MfeI site in Intermediate 1	AmpR, GentR	This Study

Strain Name	Description	Abx Markers	Reference
XL-Gold P66 c-Myc D303 in pGEMT clone D	Harbors cloning Intermediate 2 for c-Myc D303: insertion of GentR PCR product at MfeI site in Intermediate 1	AmpR, GentR	This Study
XL-Gold P66 c-Myc E334 in pGEMT clone B	Harbors cloning Intermediate 2 for c-Myc E334: insertion of GentR PCR product at MfeI site in Intermediate 1	AmpR, GentR	This Study
XL-Gold P66 c-Myc K487 in pGEMT clone B	Harbors cloning Intermediate 2 for c-Myc K487: insertion of GentR PCR product at MfeI site in Intermediate 1	AmpR, GentR	This Study
XL-Gold P66 c-Myc C-term in pGEMT C	Harbors cloning Intermediate 2 for c-Myc C-term: insertion of GentR PCR product at MfeI site in Intermediate 1	AmpR, GentR	This Study
XL-Gold P66 c-Myc E33 in pGEMT clone B	Harbors cloning final construct for c-Myc E33: BspHI digestion removed AmpR from Intermediate 2	GentR	This Study
XL-Gold P66 c-Myc T187 in pGEMT clone A	Harbors cloning final construct for c-Myc T187: BspHI digestion removed AmpR from Intermediate 2	GentR	This Study
XL-Gold P66 c-Myc D303 in pGEMT clone A	Harbors cloning final construct for c-Myc D303: BspHI digestion removed AmpR from Intermediate 2	GentR	This Study
XL-Gold P66 c-Myc E334 in pGEMT clone B	Harbors cloning final construct for c-Myc E334: BspHI digestion removed AmpR from Intermediate 2	GentR	This Study
XL-Gold P66 c-Myc K487 in pGEMT clone A	Harbors cloning final construct for c-Myc K487: BspHI digestion removed AmpR from Intermediate 2	GentR	This Study
XL-Gold P66 c-Myc C-term in pGEMT clone A	Harbors cloning final construct for c-Myc C-term: BspHI digestion removed AmpR from Intermediate 2	GentR	This Study

Supplementary Table S2. *Borrelia burgdorferi* strains used in this study.

Note that the multiplex PCR protocol is optimized for the plasmid content of strains in the B31 A3 background. Although it was also applied to the HB19 and B313 backgrounds, it may not detect all plasmids.

Strain Name	Description	Genomic *B. burgdorferi* Plasmids Notes	Abx Markers	Reference
B31 A3 (WT)	Infectious wild-type B31 A3 *B. burgdorferi* strain	Missing: Cp9 (C)		(Elias et al., 2002)
B31 A3 KO4 C3-14 (Δ*p66*)	Derivative of B31 A3 with *p66* replaced with a kanamycin resistance cassette	Missing: Cp9 (C)	KanaR	(Ristow et al., 2012)
B31 A3 KO4 C3-14 + pBSV2G P66 C2-17 A-4 (*p66*^{cp})	Derivative of B31 A3 KO4 C3-14 with *p66* complemented *in trans* on pBSV2G	Missing: Cp9 (C), Lp25 (E)	KanaR, GentR	This Study
B31 A3 KO4 C3-14 + pBSV2G P66 myc E33 A-2 (B31 A3-E33^{cp})	Derivative of B31 A3 KO4 C3-14 with *p66,* with a c-Myc tag (immediately C-terminal to E33), complemented *in trans* on pBSV2G	Missing: Cp9 (C), Lp25 (E)	KanaR, GentR	This Study
B31 A3 KO4 C3-14 + pBSV2G P66 myc T187 B-3 (B31 A3-T187^{cp})	Derivative of B31 A3 KO4 C3-14 with *p66,* with a c-Myc tag (immediately C-terminal to T187), complemented *in trans* on pBSV2G	Missing: Cp9 (C), Lp25 (E)	KanaR, GentR	This Study
B31 A3 KO4 C3-14 + pBSV2G P66 myc D303 C-4 (B31 A3-D303^{cp})	Derivative of B31 A3 KO4 C3-14 with *p66,* with a c-Myc tag (immediately C-terminal to D303), complemented *in trans* on pBSV2G	Missing: Cp9 (C), Lp25 (E)	KanaR, GentR	This Study
B31 A3 KO4 C3-14 + pBSV2G P66 myc E334 C-4 (B31 A3-E334^{cp})	Derivative of B31 A3 KO4 C3-14 with *p66,* with a c-Myc tag (immediately C-terminal to E334), complemented *in trans* on pBSV2G	Missing: Cp9 (C), Lp25 (E)	KanaR, GentR	This Study
B31 A3 KO4 C3-14 + pBSV2G P66 myc K487 3B-3 (B31 A3-K487^{cp})	Derivative of B31 A3 KO4 C3-14 with *p66,* with a c-Myc tag (immediately C-terminal to K487), complemented *in trans* on pBSV2G	Missing: Cp9 (C), Lp25 (E)	KanaR, GentR	This Study
B31 A3 KO4 C3-14 + pBSV2G P66 myc N580 B-2 (B31 A3-N580^{cp})	Derivative of B31 A3 KO4 C3-14 with *p66,* with a c-Myc tag (immediately C-terminal to N580), complemented *in trans* on pBSV2G	Missing: Cp9 (C), Lp25 (E)	KanaR, GentR	This Study
B31 A3 KO4 C3-14 + pBSV2G P66 myc C-term B-2 (B31 A3-C-term^{cp})	Derivative of B31 A3 KO4 C3-14 with *p66,* with a c-Myc tag at the C-terminus, complemented *in trans* on pBSV2G	Missing: Cp9 (C), Lp25 (E)	KanaR, GentR	This Study
B31 A3 P66 c-Myc E33^{cc} (B31 A3-E33^{cc})	Derivative of B31 A3 KO4 C3-14 with c-Myc tagged *p66* (tag immediately C-terminal to E33) complemented *in cis* to the chromosome	Missing: Cp9 (C)	GentR	This Study
P66 c-Myc T187^{cc} (B31 A3-T187^{cc})	Derivative of B31 A3 KO4 C3-14 with c-Myc tagged *p66* (tag immediately C-terminal to T187) complemented *in cis* to the chromosome	Missing: Cp9 (C)	GentR	This Study
P66 c-Myc D303^{cc} (B31 A3-D303^{cc})	Derivative of B31 A3 KO4 C3-14 with c-Myc tagged *p66* (tag immediately C-terminal to D303) complemented *in cis* to the chromosome	Missing: Cp9 (C)	GentR	This Study

**Strain Name**	**Description**	**Genomic *B. burgdorferi* Plasmids Notes**	**Abx Markers**	**Reference**
P66 c-Myc E334^{cc} (B31 A3-E334^{cc})	Derivative of B31 A3 KO4 C3-14 with c-Myc tagged *p66* (tag immediately C-terminal to E334) complemented *in cis* to the chromosome	Missing: Cp9 (C)	GentR	This Study
P66 c-Myc K487^{cc} (B31 A3-K487^{cc})	Derivative of B31 A3 KO4 C3-14 with c-Myc tagged *p66* (tag immediately C-terminal to K487) complemented *in cis* to the chromosome	Missing: Cp9 (C)	GentR	This Study
P66 c-Myc C-term^{cc} (B31 A3-C-term^{cc})	Derivative of B31 A3 KO4 C3-14 with c-Myc tagged *p66* (C-terminal tag) complemented *in cis* to the chromosome	Missing: Cp9 (C)	GentR	This Study
B313 (B313-WT)	Non-infectious wild-type B313 *B. burgdorferi* strain	Missing: Cp32-8 (L), Cp32-6 (M), Cp32-9 (N), Cp9 (C), CP32-7 (O), Lp21 (U), Lp28-3 (H), Lp38 (J), Lp28-1 (F), Lp25 (E), Lp36 (K), Lp56 (Q), Lp54 (A), Lp5 (T), Lp28-2 (G), Lp28-4 (I)		(Sadziene et al., 1993)
B313 + pBSV2G P66 myc E33 C-2 (B313-E33)	Derivative of B313 with *p66* with a c-Myc tag (immediately C-terminal to E33) encoded on pBSV2G	Missing: Same as B313 WT	GentR	This Study
B313 + pBSV2G P66 myc T187 B-3 (B313-T187)	Derivative of B313 with *p66,* with a c-Myc tag (immediately C-terminal to T187) encoded on pBSV2G	Missing: Same as B313 WT	GentR	This Study
B313 + pBSV2G P66 myc D303 B-4 (B313-D303)	Derivative of B313 with *p66,* with a c-Myc tag (immediately C-terminal to D303) encoded on pBSV2G	Missing: Same as B313 WT	GentR	This Study
B313 + pBSV2G P66 myc E334 C-1 (B313-E334)	Derivative of B313 with *p66,* with a c-Myc tag (immediately C-terminal to E334) encoded on pBSV2G	Missing: Same as B313 WT	GentR	This Study
B313 + pBSV2G P66 myc K487 A-5 (B313-K487)	Derivative of B313 with *p66,* with a c-Myc tag (immediately C-terminal to K487) encoded on pBSV2G	Missing: Same as B313 WT	GentR	This Study
B313 + pBSV2G P66 myc N580 B-1 (B313-N580)	Derivative of B313 with *p66,* with a c-Myc tag (immediately C-terminal to N580) encoded on pBSV2G	Missing: Same as B313 WT	GentR	This Study
B313 + pBSV2G P66 myc C-term B-2 (B313-C-term)	Derivative of B313 with *p66,* with a c-Myc tag at the C-terminus encoded on pBSV2G	Missing: Same as B313 WT	GentR	This Study

Strain Name	Description	Genomic *B. burgdorferi* Plasmids Notes	Abx Markers	Reference
B313 + pBSV2G P66 C2-17 B-4 (*p66^{cp}*)	Derivative of B313 with *p66* complemented from pBSV2G	Missing: Same as B313 WT	GentR	This Study
HB19 (WT)	Non-infectious, high passage, wild-type HB19 *B. burgdorferi* strain	Only detected plasmids Lp54 (A), Cp26 (B), and Lp17 (D)		(Coburn et al., 1993)
HB19 KO4 3-8B (Δ*p66*)	Derivative of HB19 with *p66* replaced with a kanamycin resistance cassette	Same HB19 WT	KanaR	(Coburn and Cugini, 2003)
HB19 + pBSV2G clone A6 (WT + vector)	Derivative of HB19 WT with pBSV2G empty vector complemented *in trans*	Same HB19 WT	GentR	This Study
HB19 KO4 + pBSV2G clone A8 (Δ*p66* + vector)	Derivative of HB19 KO4 3-8B with pBSV2G empty vector complemented *in trans*	Same HB19 WT	KanaR, GentR	This Study
HB19 KO4 + pBSV2G p66 clone 5 (HB19-P66^{cp})	Derivative of HB19 KO4 3-8B with *p66* complemented *in trans* on pBSV2G	Same HB19 WT	KanaR, GentR	(Ristow et al., 2015)
HB19 KO4 3-8B + P66 myc E33 clone B8 (HB19-E33)	Derivative of HB19 3-8B with *p66,* with a c-Myc tag (immediately C-terminal to E33), complemented *in trans* on pBSV2G	Same HB19 WT	KanaR, GentR	This Study
HB19 KO4 3-8B + P66 myc T187 clone B8 (HB19-T187)	Derivative of HB19 3-8B with *p66,* with a c-Myc tag (immediately C-terminal to T187), complemented *in trans* on pBSV2G	Same HB19 WT	KanaR, GentR	This Study
HB19 KO4 3-8B + P66 myc D303 clone B8 (HB19-D303)	Derivative of HB19 3-8B with *p66,* with a c-Myc tag (immediately C-terminal to D303), complemented *in trans* on pBSV2G	Same HB19 WT	KanaR, GentR	This Study
HB19 KO4 3-8B + P66 myc E334 clone B8 (HB19-E334)	Derivative of HB19 3-8B with *p66,* with a c-Myc tag (immediately C-terminal to E334), complemented *in trans* on pBSV2G	Same HB19 WT	KanaR, GentR	This Study
HB19 KO4 3-8B + P66 myc K487 clone B8 (HB19-K487)	Derivative of HB19 3-8B with *p66,* with a c-Myc tag (immediately C-terminal to K487), complemented *in trans* on pBSV2G	Same HB19 WT	KanaR, GentR	This Study
HB19 KO4 3-8B + P66 myc C-term clone B8 (HB19-Cterm)	Derivative of HB19 3-8B with *p66,* with a c-Myc tag at the C-terminus, complemented *in trans* on pBSV2G	Same HB19 WT	KanaR, GentR	This Study

Supplementary Table S3. Primer sequences for cloning a c-Myc tags in different regions of *p66* by Gibson assembly

Primer Name	Sequence (5'-3')	Description
pBSV2G.obb0602u	GAT TAC GAA TTC GAG CTC GGT TGA TCT TAG TAG TTC GGA TCT C	Overlaps with pBSV2G and *obb602* within the *p66* insert
pBSV2G.obb0604m	CAG TGC CAA GCT TGC ATG CCA ACC TTA ACA ATA CCT TTT GTA CTG	Overlaps with pBSV2G and *obb604* within the *p66* insert
Over E33 1	GAC GAG GAA CAA AAA CTT ATT TCT GAA GAA GAT CTG CTT GTT CCT GGG TTT GAA AAC	Pairs with pBSV2G.obb0602u to insert c-Myc at E33
Over E33 2	GAA CAA GCA GAT CTT CTT CAG AAA TAA GTT TTT GTT CCT CGT CCA TAT CTA ATC TG	Pairs with pBSV2G.obb0604m to insert c-Myc at E33
Over Integrin T87 1	GAC ACT GAA CAA AAA CTT ATT TCT GAA GAA GAT CTG CCA TAC AAT AAA ACA TAT CAA GG	Pairs with pBSV2G.obb0602u to insert c-Myc at T187
Over Integrin T87 2	GTA TGG CAG ATC TTC TTC AGA AAT AAG TTT TTG TTC AGT GTC TTT GTC ATT CTC TTG	Pairs with pBSV2G.obb0604m to insert c-Myc at T188
Over D303 1	GAA TAG ATG AAC AAA AAC TTA TTT CTG AAG AAG ATC TGC CTT TTG CAA GCG ATT TTT C	Pairs with pBSV2G.obb0602u to insert c-Myc at D303
Over D303 2	CAA AAG GCA GAT CTT CTT CAG AAA TAA GTT TTT GTT CAT CTA TTC CAA AAT CAG TTC C	Pairs with pBSV2G.obb0604m to insert c-Myc at D303
Over E335 1	GCT GAA GAA CAA AAA CTT ATT TCT GAA GAA GAT CTG ATA TTT GAT CCA AAT GGC AAT GC	Pairs with pBSV2G.obb0602u to insert c-Myc at E334
Over E335 2	CAA ATA TCA GAT CTT CTT CAG AAA TAA GTT TTT GTT CTT CAG CTT TTT TGT TAG GAT CTG	Pairs with pBSV2G.obb0604m to insert c-Myc at E334
Over K487 1	CAA AGG AAC AAA AAC TTA TTT CTG AAG AAG ATC TGA CCA CAA CCC CTA ATC TGA CAT TTG	Pairs with pBSV2G.obb0602u to insert c-Myc at K487
Over K487 2	GTG GTC AGA TCT TCT TCA GAA ATA AGT TTT TGT TCC TTT GTG CTT GTT GAA CTT TGT TC	Pairs with pBSV2G.obb0604m to insert c-Myc at K487
Over N580 1	CAA ATA ATG AAC AAA AAC TTA TTT CTG AAG AAG ATC TGG CTG CTA TTG GAA GTG C	Pairs with pBSV2G.obb0602u to insert c-Myc at N580
Over N580 2	GCA GCC AGA TCT TCT TCA GAA ATA AGT TTT TGT TCA TTA TTT GTG TTT TTA TTA GC	Pairs with pBSV2G.obb0604m to insert c-Myc at N580
Over C-term 1	GAA GCG AAC AAA AAC TTA TTT CTG AAG AAG ATC TGT AAC AGC AAA AGA AGG GCT TTG GCC	Pairs with pBSV2G.obb0602u to insert c-Myc at C-terminus
Over C-term 2	CTG TTA CAG ATC TTC TTC AGA AAT AAG TTT TTG TTC GCT TCC GCT GTA GGC TAT TTT G	Pairs with pBSV2G.obb0604m to insert c-Myc at C-terminus
aacCIMfe	CAA TTG CGA TCT CGG CTT GAA CG	Pairs with flgBMfe to amplify the GentR cassette with flanking MfeI sites (Ristow et al., 2012)
flgBMfe	CAA TTG TAA TAC CCG AGC TTC AAG GAA G	Pairs with aacCIMfe to amplify the GentR cassette with flanking MfeI sites (Ristow et al., 2012)

Supplementary Table S4. Primers used to sequence *p66* locus in mutants.

Primer Name	Sequence (5'-3')	Reference
OLCR01	ATG CCT TGA TTA CGC TGG AG	(Ristow et al., 2012)
OLCR02	TGC TCC CCA GTT ACA GTT CC	(Ristow et al., 2012)
OLCR03	AAC TTG AAT CTG ATG GTT ATG AAG C	(Ristow et al., 2012)
OLCR04	AAC CTC ATC ATC GCT AGC AC	(Ristow et al., 2012)
OLCR05	ATT TGC AAG GAA AGA AAT ATA AGG	(Ristow et al., 2012)
OLCR06	TGT TGA AAT GGA TGC TAT TGG	(Ristow et al., 2012)
OLCR07	TTG AAG ATG CAA TGA AAC TCG	(Ristow et al., 2012)
OLCR08	GGG ATT ATA AAT GGA TTA GGA TGG	(Ristow et al., 2012)
OLCR09	TGC ATT TTC AAC AGG AGC AA	(Ristow et al., 2012)
OLCR10	TCA AGA GAA TGA CAA AGA CAC TCC	(Ristow et al., 2012)
OLCR11	TTT CAA ACC CAG GAA CAA GC	(Ristow et al., 2012)
OLCR12	AGC AAT CCT GTT GCT AAA ATG	(Ristow et al., 2012)
OLCR12 Rev Comp	CAT TTT AGC AAC AGG ATT GCT	This Study
OLCR13	TTA ATC TTG ATA TTG CAA CAA TGC	(Ristow et al., 2012)
OLCR14	GGA TTA TCT CTC CGG GCT TC	(Ristow et al., 2012)
OJLC29	ATA AAG GAT TCC TTG ATA TGT TTT ATT	(Ristow et al., 2012)
OJLC32	CAC TAA AAG CGG AAG GCA AAA AAG GC	(Ristow et al., 2012)
OJLC35	CGC CCA GGA TTC TTT TTC ACC GGT A	(Ristow et al., 2012)
ONN660	AAT ATG GCC TTG AAT TTT TAC CTA ATA	(Ristow et al., 2012)
oMCW1	TTT AAA ATT GAC GTG GGA GAT	This study

Supplementary Table S5. Percentage of P66 Degraded in Relation to Flagellin for B31 A3 c-Myc P66^{cp} strains.

Imagelab (5.2.1) was used to quantify the density of each flagellin band relative to the 0 µg ml^{-1} proteinase K flagellin band for each *B. burgdorferi* strain. Similarly, the density of the c-Myc band was quantified relative to the 0 µg ml^{-1} proteinase K concentration c-Myc band. In contrast, densitometry was performed on the P66 band for the WT and *p66^{cp}* control strains. The percent decrease of P66 was calculated by subtracting the relative P66 (or c-Myc) band density from the relative flagellin band density, then dividing by the relative flagellin band density $(\frac{(relative\ flagellin\ density - relative\ P66\ (or\ c-Myc)\ density)}{relative\ flagellin\ density})$ x 100.

Proteinase K Concentration (µg ml^{-1})	Percent decrease of P66								
	WT	*p66^{cp}*	E33	T187	D303	E334	K487	N580	C-term
0	0%	0%	0%	0%	0%	0%	0%	0%	0%
1	6%	37%	57%	-58%	95%	38%	76%	-24%	19%
10	97%	87%	60%	84%	94%	92%	92%	-32%	85%
50	99%	94%	75%	98%	88%	88%	93%	-27%	95%

Supplementary Table S6. Statistical comparison of growth between *B. burgdorferi* B31 A3 c-Myc P66^{cp} strains in BSKII and BSKII + vancomycin (1 µg ml^{-1}).

GraphPad Prism 9.2.0 was used to calculate statistically significant differences in slope by simple linear regression for the different strains and treatment groups.

	BSKII		BSKII + vancomycin	
	WT	Δ*p66*	WT	Δ*p66*
WT		ns		p<0.0001
Δ*p66*	ns		p<0.0001	
p66^{cp}	ns	ns	ns	p<0.0001
E33	ns	ns	p<0.0001	p<0.0001
T187	ns	ns	ns	p<0.0001
D303	ns	ns	p<0.0001	p<0.05
E334	ns	ns	ns	p<0.0001
K487	ns	ns	ns	p<0.0001
N580	p<0.005	p<0.005	p<0.05	p<0.0001
C-term	ns	ns	ns	p<0.0001

Supplementary Table S7. Statistical comparison of growth between B31 A3 c-Myc P66^{cc} strains in BSKII and BSKII + vancomycin (1 µg ml^{-1}).

GraphPad Prism 9.2.0 was used to calculate statistically significant differences in slope by simple linear regression for the different strains and treatment groups.

	BSKII		BSKII + vancomycin	
	WT	Δ*p66*	WT	Δ*p66*
WT		ns		p<0.05
Δ*p66*	ns		p<0.05	
E33	ns	ns	p<0.05	p<0.05
T187	ns	ns	ns	p<0.05
D303	ns	ns	p<0.05	p<0.05
E334	ns	ns	ns	p<0.05
K487	ns	ns	ns	p<0.05
C-term	p<0.05	ns	ns	p<0.05

Supplementary Table S8. Percentage of P66 Degraded in Relation to Flagellin for B31 A3 c-Myc $P66^{cc}$ strains.

Image Lab (6.0) was used to quantify the density of each flagellin band relative to the 0 µg ml^{-1} Proteinase K flagellin band for each *B. burgdorferi* strain. Similarly, the density of the P66 (or c-Myc) band was quantified relative to the 0 µg ml^{-1} Proteinase K P66 (or c-Myc) band as shown in Supplementary Figure S5. The percent decrease of P66 (or c-Myc) was calculated by subtracting the relative P66 (or c-Myc) band density from the relative flagellin band density, then dividing by the relative flagellin band density ($\frac{(relative\ flagellin\ density - relative\ P66\ (or\ c-Myc)\ density)}{relative\ flagellin\ density}$) x 100.

Proteinase K Concentration (µg ml^{-1})	Percent decrease of P66						
	WT	E33	T187	D303	E334	K487	C-term
0	0%	0%	0%	0%	0%	0%	0%
0.3	-20%	-16%	-33%	39%	-27%	29%	-31%
1	-24%	36%	-2%	93%	-23%	42%	53%
3	-27%	53%	42%	98%	93%	75%	60%
10	47%	95%	70%	96%	88%	95%	83%
30	69%	97%	75%	96%	83%	95%	91%

Supplementary Table S9. Total numbers of culture-positive tissues from mice inoculated with B31 A3 and derivative strains at a dose of $1x10^5$ spirochetes/mouse.

Culture positivity for each group of 5 mice is shown at 1 week post-harvest. Values that changed upon further incubation are indicated in bold and underlined. Data for T187 and K487 were monitored only for 1 week and are in gray.

	Culture Positive/Total Mice							
Inoculated Strain	Blood	Bladder	Heart	Ankle	Knee	Skin	Ear	Total Mice
WT	4/5	5/5	5/5	5/5	**1/5** → **5/5**	5/5	**4/5** → **5/5**	5/5
Δ*p66*	0/5	0/5	0/5	0/5	0/5	0/5	0/5	0/5
E33 clone A3	0/5	0/5	0/5	0/5	0/5	1/5	0/5	1/5
E33 clone B17	0/5	0/5	0/5	0/5	0/5	0/5	0/5	0/5
T187 clone A2	0/5	3/5	3/5	3/5	3/5	3/5	3/5	3/5
T187 clone B5	0/5	2/5	3/5	2/5	3/5	3/5	3/5	3/5
D303 clone A30	0/5	0/5	0/5	0/5	0/5	1/5	1/5	1/5
D303 clone B20	0/5	0/5	0/5	0/5	0/5	1/5	0/5	1/5
E334 clone A1	0/5	**2/5** → **4/5**	**1/5** → **4/5**	1/5	4/5	4/5	4/5	4/5
E334 clone B8	0/5	3/5	**1/5** → **2/5**	0/5	3/5	3/5	3/5	3/5
K487 clone B17	0/5	5/5	5/5	5/5	5/5	5/5	5/5	5/5
K487 clone C16	0/5	5/5	5/5	5/5	5/5	5/5	5/5	5/5
C-term clone A1	1/5	5/5	5/5	4/5	4/5	5/5	5/5	5/5
C-term clone C57	1/5	4/5	5/5	4/5	5/5	5/5	5/5	5/5

Supplementary Table S10. Total numbers of culture-positive tissues from mice inoculated with c-Myc E33^{cc} and c-Myc D303^{cc} at various doses.

* One mouse was lost to an accident in the animal facility.

Inoculated Strain	Spirochete Dose	Culture Positive/Total Mice								ID_{50} Value
		Blood	Bladder	Heart	Ankle	Knee	Skin	Ear	Total Mice	
E33 clone A3	$1x10^3$	0/5	0/5	0/5	0/5	0/5	0/5	0/5	0/5	$6.76x10^5$
	$1x10^5$	0/5	0/5	0/5	0/5	0/5	0/5	0/5	0/5	
	$1x10^7$	0/5	0/5	2/5	0/5	0/5	3/5	1/5	3/5	
	$1x10^9$	0/5	1/5	3/5	1/5	1/5	5/5	5/5	5/5	
E33 clone B17	$1x10^3$	0/5	0/5	0/5	0/5	0/5	0/5	0/5	0/5	$6.76x10^5$
	$1x10^5$	0/5	0/5	0/5	0/5	0/5	0/5	0/5	0/5	
	$1x10^7$	0/5	0/5	2/5	1/5	1/5	3/5	2/5	3/5	
	$1x10^9$	0/5	1/5	4/5	1/5	1/5	5/5	4/5	5/5	
D303 clone A30	$1x10^3$	0/5	0/5	0/5	0/5	0/5	0/5	0/5	0/5	$3.16x10^5$
	$1x10^5$	0/5	0/5	0/5	0/5	0/5	0/5	0/5	0/5	
	$1x10^7$ *	1/4	0/4	1/4	2/4	2/4	4/4	1/4	4/4	
	$1x10^9$	1/5	2/5	5/5	4/5	5/5	5/5	4/5	5/5	
D303 clone B20	$1x10^3$	0/5	0/5	0/5	0/5	0/5	0/5	0/5	0/5	$1.48x10^5$
	$1x10^5$	0/5	0/5	2/5	0/5	0/5	2/5	0/5	2/5	
	$1x10^7$	0/5	0/5	4/5	1/5	5/5	5/5	5/5	5/5	
	$1x10^9$	0/5	3/5	4/5	1/5	5/5	5/5	5/5	5/5	

References

Coburn, J., and Cugini, C. (2003). Targeted mutation of the outer membrane protein P66 disrupts attachment of the Lyme disease agent, *Borrelia burgdorferi*, to integrin αvβ3. *Proceedings of the National Academy of Sciences* 100(12)**,** 7301-7306.

Coburn, J., Leong, J.M., and Erban, J.K. (1993). Integrin alpha IIb beta 3 mediates binding of the Lyme disease agent *Borrelia burgdorferi* to human platelets. *Proceedings of the National Academy of Sciences* 90(15)**,** 7059-7063.

Elias, A.F., Stewart, P.E., Grimm, D., Caimano, M.J., Eggers, C.H., Tilly, K., et al. (2002). Clonal Polymorphism of *Borrelia burgdorferi* Strain B31 MI: Implications for Mutagenesis in an Infectious Strain Background. *Infection and Immunity* 70(4)**,** 2139-2150. doi: 10.1128/iai.70.4.2139-2150.2002.

Ristow, L.C., Bonde, M., Lin, Y.P., Sato, H., Curtis, M., Wesley, E., et al. (2015). Integrin binding by *Borrelia burgdorferi* P66 facilitates dissemination but is not required for infectivity. *Cellular Microbiology* 17(7)**,** 1021-1036.

Ristow, L.C., Miller, H.E., Padmore, L.J., Chettri, R., Salzman, N., Caimano, M.J., et al. (2012). The beta(3)-integrin ligand of *Borrelia burgdorferi* is critical for infection of mice but not ticks. *Molecular Microbiology* 85(6)**,** 1105-1118. doi: 10.1111/j.1365-2958.2012.08160.x.

Sadziene, A., Wilske, B., Ferdows, M.S., and Barbour, A.G. (1993). The cryptic *ospC* gene of *Borrelia burgdorferi* B31 is located on a circular plasmid. *Infection and Immunity* 61(5)**,** 2192-2195.
